# Role of fourteen XRE-DUF397 pairs from *Streptomyces coelicolor* as regulators of antibiotic production and differentiation. New players in a complex regulatory network

**DOI:** 10.3389/fmicb.2023.1217350

**Published:** 2023-07-10

**Authors:** Carolina Riascos, Ana Martínez-Carrasco, Margarita Díaz, Ramón I. Santamaría

**Affiliations:** Instituto de Biología Funcional y Genómica (IBFG), Departamento de Microbiología y Genética, Consejo Superior de Investigaciones Científicas (CSIC), Universidad de Salamanca (USAL), Salamanca, Spain

**Keywords:** *Streptomyces*, antibiotic production, xenobiotic regulator response (XRE), DUF397, morphological differentiation

## Abstract

Bacteria of the genus *Streptomyces* have a plethora of transcriptional regulators, among which the xenobiotic response element (XRE) plays an important role. In this organism, XRE regulators are often followed downstream by small proteins of unknown function containing a DUF397 domain. It has been proposed that XRE/DUF397 pairs constitute type II toxin–antitoxin (TA) systems. However, previous work carried out by our group has shown that one of these systems is a strong activator of antibiotic production in *S. coelicolor* and other *Streptomyces* species. In this work, we have studied the overexpression of fourteen XRE/DUF397 pairs present in the *S. coelicolor* genome and found that none behave as a type II TA system. Instead, they act as pleiotropic regulators affecting, in a dependent manner, antibiotic production and morphological differentiation on different culture media. After deleting, individually, six XRE/DUF397 pairs (those systems producing more notable phenotypic changes when overexpressed: SCO2246/45, SCO2253/52, SCO4176/77, SCO4678/79, SCO6236/35, and SCO7615/16), the pair SCO7615/16 was identified as producing the most dramatic differences as compared to the wild-type strain. The SCO7615/16 mutant had a different phenotype on each of the media tested (R2YE, LB, NMMP, YEPD, and MSA). In particular, on R2YE and YEPD media, a bald phenotype was observed even after 7 days, with little or no actinorhodin (ACT) production. Lower ACT production was also observed on LB medium, but the bacteria were able to produce aerial mycelium. On NMMP medium, the mutant produced a larger amount of ACT as compared with the wild-type strain.

## Introduction

*Streptomyces* species can withstand environmental instability, microbial competition, cell cycle, and secondary metabolism induction, among other events, due to the presence of a plethora of regulatory elements in their genomes permitting their adaptation to changing habitats (Chandra and Chater, 2014; Lewin et al., 2016; Shepherdson et al., 2023). Consequently, the number of regulators present is correlated with the complexity of each microorganism, where the number of regulatory proteins is higher in those species with complex cell cycles or unstable habitats. Therefore, understanding how regulatory networks function facilitates their manipulation and use for improving some of the capabilities of *Streptomyces* species (Xia et al., 2020; Sanchez de la Nieta et al., 2022; Zhang et al., 2022).

The most important and interesting aspect of bacteria from this genus is its ability to produce a wide array of secondary metabolites (Alam et al., 2022). In fact, all of the *Streptomyces* genomes analyzed can produce a high number of natural products (NPs) (a mean of 30 per specie) but only a few are synthesized under laboratory conditions. Consequently, this limitation has attracted the interest of different pharmaceutical companies since many of the metabolites generated are bioactive molecules with antibiotic, antifungal, and antitumoral properties among others (Nepal and Wang, 2019; Liu et al., 2021; Lacey and Rutledge, 2022). As a result, the development of strategies to improve the production of these NPs is of particular interest as a means to overcome the rising issue of antibiotic resistance and for producing new anti-cancer drugs (Kealey et al., 2017; Alam et al., 2022).

The strategy we have undertaken for enlightening the regulation of antibiotic production, and for developing new tools for improving production, is to add pieces in the networks from the signals that induce antibiotic production to the activated genes responsible for the biosynthesis. These signal transductions are carried out by different types of regulators among which two-component systems (TCSs) play an important role and of which have been the subject of many studies (McLean et al., 2019; Sanchez de la Nieta et al., 2022). However, there are many other unexplored regulators in *Streptomyces* sp. such as the XRE family (*X*enobiotic *R*esponse *E*lement). XRE proteins are the second most frequently occurring regulator family in bacteria. These regulators control diverse metabolic functions and are widely distributed throughout prokaryotes and eukaryotes (Ulrich et al., 2005; Lu et al., 2019; Trouillon et al., 2021). They have an N-terminal helix–turn–helix (HTH) DNA-binding domain, which can bind to the promoter of the target genes and modulate their transcription, and a highly variable C-terminal region.

In actinobacteria, a high number of the open reading frames (ORFs) encoding XRE proteins are clustered with a gene that encodes a small protein with a *D*omain of *U*nknown *F*unction identified as DUF397. It has been proposed that these XRE/DUF397 pairs are type II toxin-antitoxin (TA) systems, where the XRE protein is the antitoxin and the DUF397 is the toxin (Makarova et al., 2009; Doroghazi and Buckley, 2014).

In previous studies, we analyzed the XRE-DUF397 pair in *S. coelicolor* comprising the proteins encoded by the *SCO4441/4442* genes, denominated Scr1/Scr2. We showed that the overexpression of the XRE protein, Scr1, had a strong and positive effect on the production of different antibiotics (Santamaría et al., 2018). Interestingly, Scr2, a DUF397 protein comprising 63 amino acids, is necessary for the functionality of Scr1. Also, although the Scr1/Scr2 protein pair has been classified as a type II TA system it did not behave as one. Instead, as previously mentioned, Scr1/Scr2, when together, act as a strong and positive regulator of antibiotic production (Santamaría et al., 2018). The T/A database TADB2^1^ has identified, bioinformatically, another fourteen XRE/DUF397 pairs in the *S. coelicolor* genome, which have been proposed to be TA systems (Xie et al., 2018). These fourteen XRE-DUF397 pairs are the focus of this work.

Based on previous work on Scr1/Scr2, the initial hypothesis was that some of these fourteen XRE/DUF397 pairs could also be regulators of antibiotic production, which if confirmed would add new players to the complex regulatory network triggering antibiotic production. In the present work, we have examined the effect that the overexpression of each individual gene in these 14 protein pairs and the join couple of each XRE/DUF397 system have over antibiotic production and/or morphological differentiation on *S. coelicolor* M145. The importance of these XRE-DUF397 pairs in antibiotic production and morphological differentiation was also revealed through a study involving the deletion of the six gene pairs that were found to have the greatest effect when overexpressed.

## Materials and methods

### Strains, media, and growth conditions

*Streptomyces coelicolor* M145 (prototroph; SCP1^−^, SCP2^−^) (Hopwood et al., 1985) was the original strain used to obtain all of the XRE/DUF397 genes and to generate the deletion mutants obtained in this study (*S. coelicolor ∆SCO2246/45*, *S. coelicolor ∆SCO2253/52*, *S. coelicolor ∆SCO4678/79*, *S. coelicolor ΔSCO6236/35*, *S. coelicolor ΔSCO7615/16*). This strain and the subsequent mutants were grown on LB, PGA, MSA, NMMP, R2YE, and YEPD solid media for carrying out the phenotypic assays, R2YE was used for transformation, MSA for sporulation, and YEPD for spore quantification. *Escherichia coli* DH5α and *E. coli* ET12567 (a dam^−^ strain) were used when constructing the various plasmids and for obtaining the DNA used to transform *S. coelicolor* (Sambrook et al., 1989; McLean et al., 2019). Transformation and manipulation of *S. coelicolor* and *E. coli* DNA were performed using the method described in Green and Sambrook (2012) and Kieser et al. (2000). *Bacillus subtilis* CECT 4522 was used in the antibiogram experiments. When necessary, the medium was supplemented with the antibiotics: ampicillin (100 μg mL^−1^), kanamycin (50 μg mL^−1^), chloramphenicol (25 μg mL^−1^), or nalidixic acid (25 μg mL^−1^) for *E. coli*; and neomycin (20 μg mL^−1^) and apramycin (50 μg mL^−1^) for *S. coelicolor*.

### DNA manipulation and plasmid construction

Total genomic DNA was extracted using the modified CTAB method (hexadecyltrimethylammonium bromide) (Cafaro et al., 2011). In total, 1.5 mL of mycelium in liquid medium was collected and transferred to a 1.5 mL screw cap tube and centrifuged at 13000 rpm for 5 min and the cells were washed with a 10.3% sucrose solution. The cells were then macerated using glass beads in a lysis solution (0.3 M Sucrose; 25 mM EDTA pH 8.0; 25 mM Tris–HCl pH 8.0) and digested with 500 μL of lysozyme (3 mg mL^−1^) and RNAse (50 μg mL^−1^) at 37°C. DNA extraction was performed in 500 μL of CTAB buffer, with a chloroform extraction step, and isopropanol precipitation was carried out for 30 min at −80°C. The DNA pellet was washed with 70% ethanol, and resuspended in TE buffer, and the integrity of the genomic DNA was verified by agarose gel electrophoresis.

Plasmid isolation, restriction enzyme digests, ligation, and transformation of *E. coli* and *S. coelicolor* were carried out using the methods developed by Green and Sambrook (2012) and Kieser et al. (2000), respectively. The oligonucleotide used to amplify the genes individually or together and those used to obtain the different mutations are listed in the Supplementary Table S1. Total DNA of the *S. coelicolor* M145 was used as the template to obtain all the studied genes. For each system, three intermediate *E. coli* plasmids were constructed using plasmid pXHis1 as the backbone (Adham et al., 2001) and named pXHisXXXX and pXHisXXXX/XX (where XXXX or XXXX/XX means the SCO or SCOs amplified). Constructs were made for each gene separately as well as constructs carrying both genes (Supplementary Table S2). All genes were under the control of the xylanase promoter isolated from *S. halstedi* (Rodriguez et al., 2005). Then, for overexpression in *Streptomyces*, the gene or genes were introduced into the shuttle plasmid *E. coli-Streptomyces* pNX4 (Rodriguez et al., 2005), generating the final plasmids named pNX-XXXX or pNX-XXXX/XX (XXXX or XXXX/XX means the SCO or SCOs amplified) (Supplementary Table S2).

### Growth and antibiotic production analysis

Overexpression of the constructed plasmids was performed in *S. coelicolor* using five culture media: LB, NMMP, PGA, and R2YE. Phenotypic studies were carried out on solid media plates inoculated with 5 × 10^5^ spores added in a 5-μL drop and incubated at 30°C for several days to monitor changes in the color of the colony, in the culture medium, and morphology. Undecylprodigiosin (RED) production (red color) can be detected after 2 days as red colonies. Actinorhodin (ACT) production (blue color) can be observed as a blue halo around the colonies after 3 days of culture. All cultures were monitored photographically on each of the culture media used for 7 days. All experiments were performed in triplicate.

Antibiotic production was quantified from cultures in liquid LB medium inoculated with 4 × 10^6^ spores mL^−1^. ACT and RED antibiotic production were quantified using the spectrophotometric method described by Yepes et al. (2011). All experiments were performed in triplicate.

For the bioassay, patches of the corresponding *S. coelicolor* strains were grown during 10 days at 30°C on solid R2YE, LB, NMMP, and PGA. Then, circular sections of the patches were collected and placed on LB plates inoculated with a lawn of *B. subtilis*. The plates were first incubated at 4°C for 5 h (to allow diffusion of any produced molecules) and then at 37°C for 24 h. The inhibition halos were measured. The error bars correspond to triplicate assays.

### Mutant constructions and complementation plasmids

The CRISPR/Cas9 system, developed by Tong et al. (2015), was used to delete the two genes of the 6 systems selected (see results). The primers used to amplify and clone in the pCRISPR-Cas9 plasmid (Tong et al., 2015) the whole sgRNA guide by PCR are indicated in Supplementary Table S1, generating pCRISPR-Cas9-sgXXXX (XXXX corresponds to the SCO genes to delete in each system) (Supplementary Table S2). One kilobase (kb) on the left and one on the right of the corresponding XRE/DUF397 genes were amplified using genomic DNA. The final DNA fragment used as a template was obtained through an overlapping PCR method using the primers indicated (Supplementary Table S1). This fragment was digested with restriction enzyme NheI and introduced into the XbaI site of pCRISPR-Cas9-sgXXXX plasmids to generate the final plasmids pCRISPR-Cas9-XXXX. The plasmids generated were verified by sequencing them. Supplementary Table S2 is a list of all plasmids generated.

For each one of the XRE/DUF397 systems, the two plasmids generated, the one harboring only the sgRNA (pCRISPR-Cas9-sgXXXX) and the final plasmid with the guide and the template (pCRISPR-Cas9- XXXX), were introduced into *E. coli* ET12567 pUZ8002. Then, the plasmids were transferred to *S. coelicolor* by interspecific conjugation and by selecting Apra-resistant colonies. An empty plasmid (pCRISPR-Cas9) was used as the negative control. To induce plasmid loss, which was thermosensitive, and get the integration of the amplified DNA into the genome, the colonies obtained were grown in liquid TSB under agitation (200 rpm) at 37°C for 2 days. Apra-sensitive colonies were selected on R2YE solid media.

Genomic DNA was obtained from the *S. coelicolor* wild-type strain and from the putative M145 mutants and the correct deletion of XRE/DUF397 genes were verified by PCR using the corresponding oligonucleotides for each gene pair (Supplementary Table S1).

For the complementation analysis, both genes under their own promotor were introduced into the pKC796 integrative plasmid generating the pKCXXXX/XX plasmids (XXXX/XX corresponds to the SCOs genes of each system) (Supplementary Table S2).

## Results

### Are the XRE/DUF397 protein pairs antibiotic regulators or functional toxin/antitoxin systems?

As mentioned before, the *S. coelicolor* genome has 15 genes that encode proteins with an XRE domain that are clustered with genes encoding a small protein with a DUF397 domain (Tables 1, 2 and Supplementary Figures S1, S2). Throughout the text, these systems are referred to as XRE/DUF397.

**Table 1:** XRE proteins followed by a protein with one DUF397 domain in *Streptomyces coelicolor*.

Proteins	Number of amino acids	HTH-XRE domain	DUF5753 domain
SCO1979	286	16–72	101–280
SCO2246	258	29–79	102–253
SCO2253	284	29–80	102–279
SCO2381	278	18–71	91–263
SCO2513	277	14–67	89–264
SCO4176	280	19–71	93–268
SCO4301	279	18–71	91–268
SCO4441	295	25–78	109–287
SCO4543	283	15–72	101–277
SCO4678	282	27–78	100–277
SCO5125	287	32–82	105–282
SCO6129	303	47–99	122–297
SCO6236	291	35–86	108–286
SCO6629	277	22–73	94–272
SCO7615	290	21–72	106–274

Data obtained with InterPro (Paysan-Lafosse et al., 2023).

**Table 2:** DUF397 domain proteins preceded by a protein with one XRE domain in *Streptomyces coelicolor*.

Proteins	Number of amino acids	Consensus disorder prediction	DUF397 domain
SCO1978	91		30–82
SCO2245	69		14–65
SCO2252	73		14–64
SCO2382	65	1–26	11–65
SCO2514	96		9–29 and 33–88
SCO4177	89		2–19 and 27–81
SCO4300	89		8–26 and 26–79
SCO4442	63		10–62
SCO4542	63		6–59
SCO4679	63		8–58
SCO5124	68		11–64
SCO6128	77		7–25 and 27–76
SCO6235	81		27–77
SCO6630	67		14–64
SCO7616	89	1–31	27–63

Data obtained with InterPro (Paysan-Lafosse et al., 2023).

**Figure 1:**
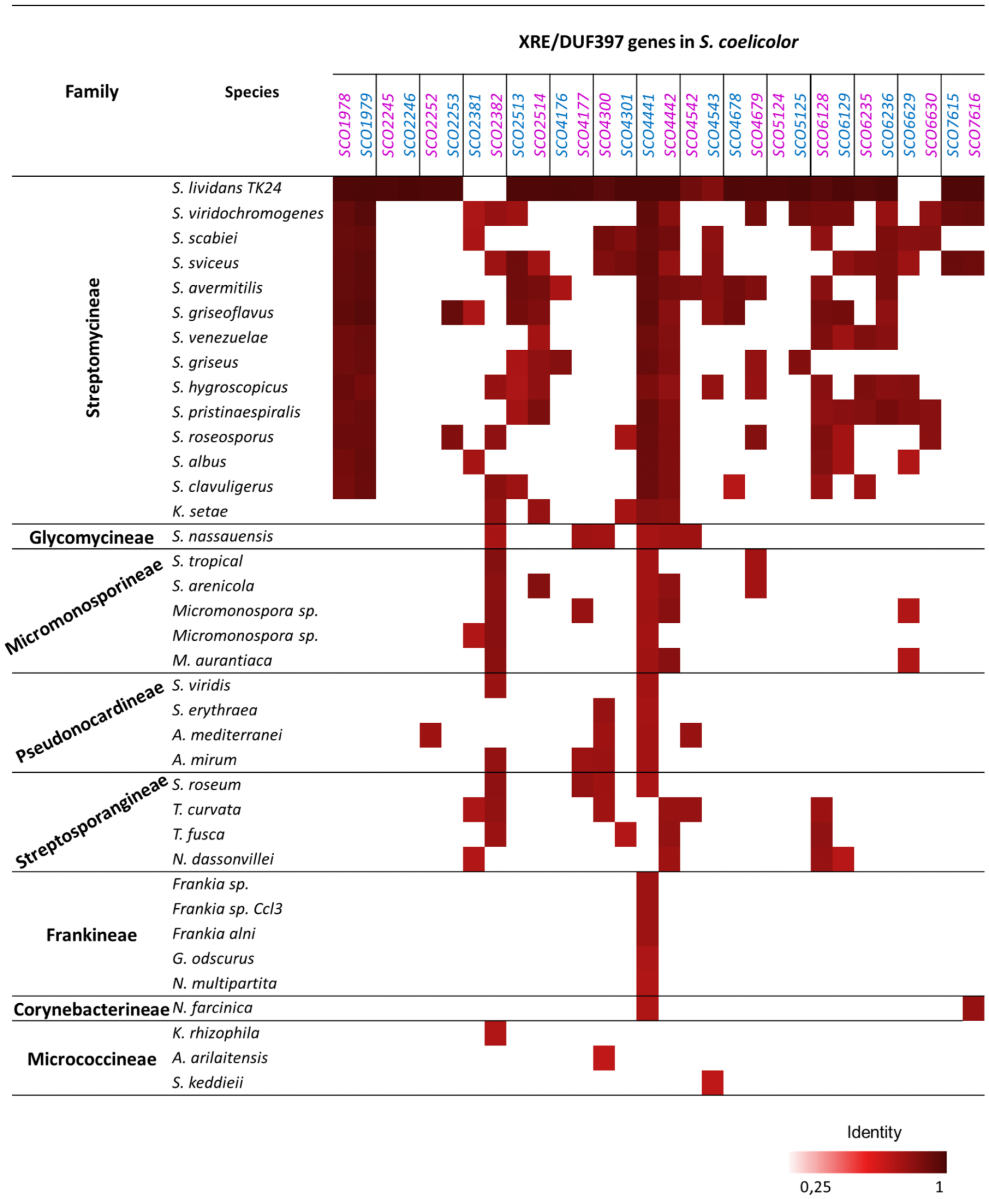
Orthologues of the 15 XRE-DUF397 genes pair from *S. coelicolor* in Actinomycetes based on an Actinoblast database search (http://streptomyces.org.uk/actinoblast/). The XRE genes are in blue and the DUF397 genes are in purple. Species included in the study: *Streptomyces lividans* TK24, *Streptomyces viridochromogenes* DSM 40736, *Streptomyces scabiei* 87.22, *Streptomyces sviceus* ATCC 29083, *Streptomyces avermitilis* MA-4680, *Streptomyces griseoflavus* Tu4000, *Streptomyces venezuelae* ATCC 10712, *Streptomyces griseus* subsp. *griseus* NBRC 13350, *Streptomyces hygroscopicus* ATCC 53653, *Streptomyces pristinaespiralis* ATCC 25486, *Streptomyces roseosporus* NRRL 15998, *Streptomyces albus* G J1074, *Streptomyces clavuligerus* ATCC 27064, *Kitasatospora setas* KM-6054; *Stackebrandtia nassauensis* DSM 44728; *Salinispora tropica* CNB-440, *Salinispora arenicola* CNS-205, *Micromonospora* sp. L5, *Micromonospora* sp. ATCC39149, *Micromonospora aurantiaca* ATCC 27029; *Saccharomonospora viridis* DSM 43017; *Saccharopolyspora erythraea* NRRL 2338; *Amycolatopsis mediterranei* U32, *Actinosynnema mirum* DSM 43827; *Streptosporangium roseum* DSM 43021, *Thermomonospora curvata* DSM 43183, *Thermobífida fusca* YX, *Nocardiopsis dassonvillei* subsp. *Dassonvillei* DSM 43111; *Frankia* sp. EAN1pec, *Frankia* sp. CcI3, *Frankia alni* ACN14a, *Geodermatophilus obscurus* DSM 43160, *Nakamurella multipartita* DSM 44233; *Nocardia farcinica* IFM 10152; *Kocuria rhizophila* DC2201, *Arthrobacter arilaitensis* Re117, *Sanguibacter keddieii* DSM 10542.

The conservation of these *S. coelicolor* systems, at the protein level, among other species of Actinobacteria, was analyzed using the *Actinoblast* database that contains more than one hundred completely sequenced actinobacterial genomes and other outsiders such as Enterobacteriaceae and Bacillaceae^2^ (Chandra and Chater, 2014). In Figure 1, it can be observed that the most conserved pair was SCO4441/4442, which was only absent in Micrococcineae and some of the Streptosporangineae species included in the comparison. Other pairs were less represented, such as systems SCO2245/2246 and SCO2252/SCO2253, which were only present in *S. coelicolor* and its relative *S. lividans*. In general, the degree of conservation was higher among *Streptomyces* spp. than with other actinomycetes. Two phylogenetic trees comprising the orthologous genes included in Figure 1 are included in Supplementary Figures S3 S4. In the XRE proteins, 6 robust groups were obtained with a frequency of ≥98 replicates while in the DUF397 only groups 5 and 6 were found with these characteristics (Supplementary Figures S3, S4). Conservation of the 15 Xre proteins and the 15 DUF397 proteins is shown in Supplementary FIgure S5.

The pair Scr1/Scr2 had been previously studied in our laboratory and was found to not behave as a toxin/antitoxin system as predicted, but rather as a potent activator of antibiotic production when overexpressed in the strain *S. coelicolor* M145 and other *Streptomyces* species (Santamaría et al., 2018). To analyze whether some of the other 14 members of this XRE/DUF397 family behaved the same as Scr1/Scr2, each gene was individually and jointly overexpressed in strain *S. coelicolor* M145.

Three multicopy plasmids were generated for each system with each gene under the control of the strong *xysAp* promoter (see Materials and Methods) (Rodriguez et al., 2005; Supplementary Table S2). The *S. coelicolor* M145 transformant strains of each construction were obtained and the effects of the overexpression of the three plasmids generated for each system was studied on solid R2YE medium and compared with the strain harboring the empty plasmid pN702Gem3 as the control (Fernandez-Abalos et al., 2003).

**Figure 2:**
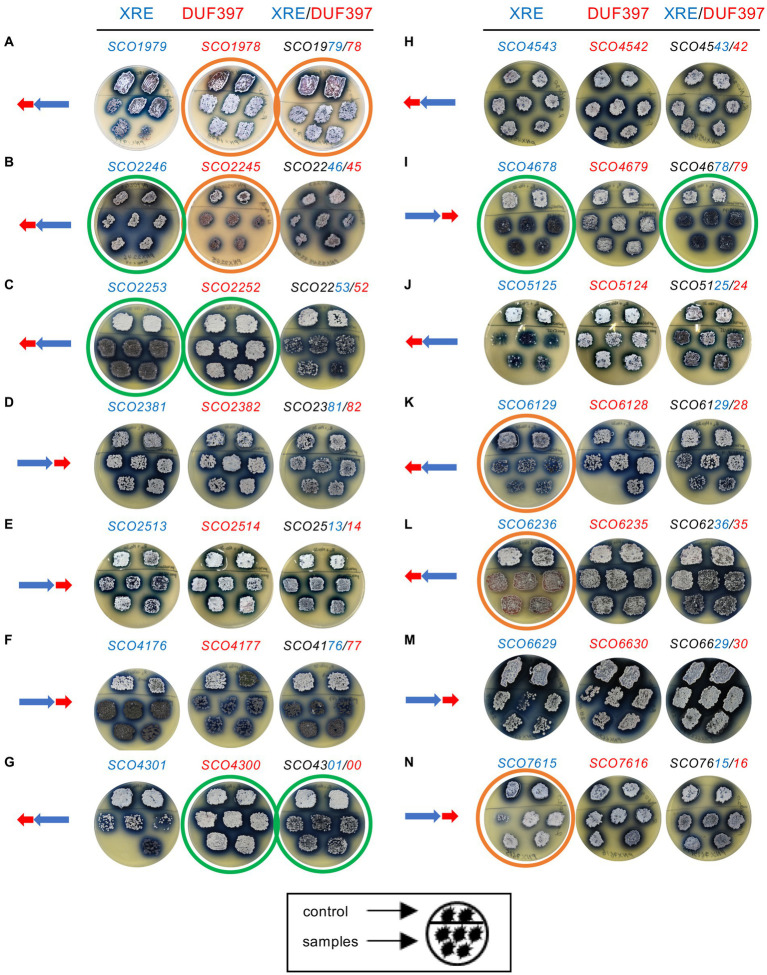
Overexpression of 14 *S. coelicolor* XRE/DUF397 systems grown on solid R2YE medium. Control: *S. coelicolor* M145/pNX702GEM3; Samples: *S. coelicolor* M145 harboring the corresponding plasmid overexpressing the gene/s indicated in the upper part of the Petri dishes. On the left of each column, a scheme of the order of genes in the genome of S. coelicolor is shown. **(A)** SCO1979/78 system; **(B)** SCO2246/45 system; **(C)** SCO2253/52 system; **(D)** SCO2381/82 system; **(E)** SCO2513/14 system; **(F)** SCO4176/77 system; **(G)** SCO4301/00 system; **(H)** SCO4543/42 system; **(I)** SCO4678/79 system; **(J)** SCO5125/24 system; **(K)** SCO6129/28 system; **(L)** SCO6236/35 system; **(M)** SCO6629/30 system; **(N)** SCO7615/16 system.

None of the *S. coelicolor* strains overexpressing the 14 putative toxins (DUF397 proteins) showed a clear negative effect on *S. coelicolor* viability, suggesting that these proteins did not produce toxic effects under the conditions used (Figures 2A–N). These results contradict the possibility of these pairs functioning as TA systems in the same way as Scr1/Scr2. However, on this medium, almost all systems exhibited differences, compared with the control, of greater or less intensity in the patterns of differentiation and/or production of ACT when at least one of their genes was overexpressed. Only systems SCO4543/42 and SCO6629/30 did not present appreciable differences with the control strain (Figures 2H–M).

Since the main interest of this study was to identify those systems involved in the regulation of antibiotic production, we selected for further study six of the systems showing higher or lower production of ACT compared to the control. Those with a drastic phenotypic alteration in morphological differentiation were also selected. The systems chosen for further study were: SCO2246/45, SCO2253/52, SCO4176/77, SCO4678/79, SCO6236/35, and SCO7615/16. So, the overexpression of the DUF gene (*SCO2245*) in pair SCO2246/45 showed a developmental delay and decreased ACT production. However, the overexpression of the XRE gene (*SCO2246*) showed an increase in ACT production (Figure 2B). In system SCO2253/52, the overexpression of the XRE (*SCO2253*) gene and both XRE/DUF genes showed a drastic developmental delay and the overexpression of the elements separately showed a slight increase in ACT production (Figure 2C). In system SCO4176/77, the separate overexpression of both genes, as well as their join overexpression, showed a clear bald phenotype but ACT production was the same as the control (Figure 2F). As for SCO4678/79, the individual overexpression of the XRE gene (*SCO4678*) and the two genes together, XRE and DUF, produced a bald phenotype and higher ACT production (Figure 2I). Conversely, for SCO6236/35, the overexpression of the XRE gene (*SCO6236*) produced a drastic reduction in ACT production. Additionally, a small delay in differentiation was also observed with the overexpression of this gene separately and the two together (Figure 2L). For system SCO7615/16, the overexpression of the XRE gene (*SCO7615*) led to lower ACT production, and the differentiation was delayed when both genes were overexpressed (Figure 2N).

### XRE/DUF397 systems are pleiotropic regulators whose effects are culture media dependent

**Figure 3:**
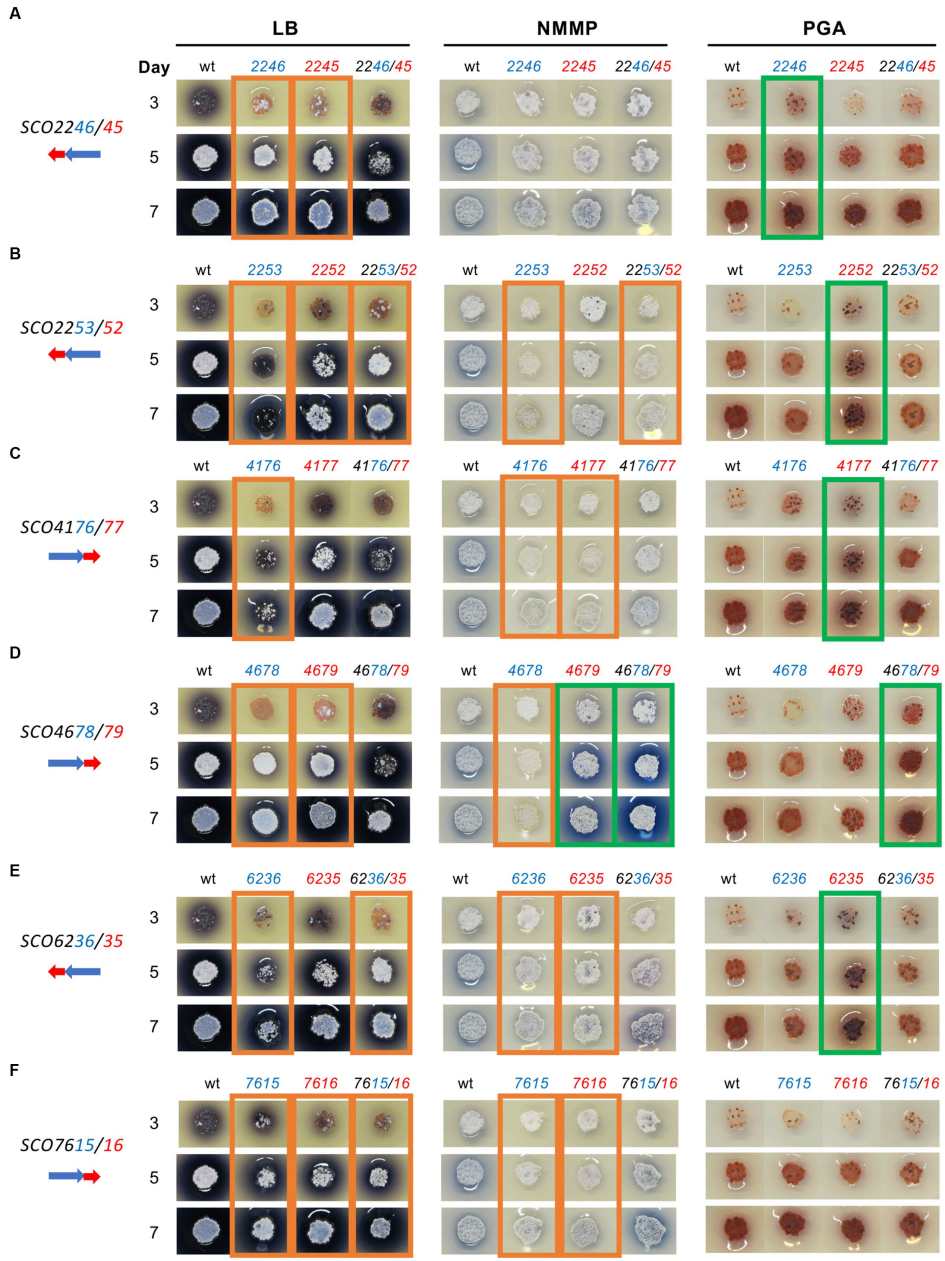
Overexpression of six *S. coelicolor* XRE/DUF397 systems on three solid media: LB, NMMP, and PGA. *S. coelicolor* M145 harboring the corresponding plasmid overexpressing the gene/s indicated in the upper part of the columns in the image. On the left, a scheme of the order of the genes in the genome of *S. coelicolor* is shown; Blue: XRE genes; Red: DUF397 genes. **(A)** *SCO2246/45* system; **(B)** *SCO2253/52* system; **(C)** *SCO4176/77* system; **(D)** *SCO4678/79* system; **(E)** *SCO6236/35* system; **(F)** *SCO7615/16* system. wt: *S. coelicolor* M145/pNX702GEM3. Ο Green, a strong increase in ACT or RED production; Ο Orange, a strong decrease in ACT or RED production.

To explore if the overexpression of the six selected systems (SCO2246/45, SCO2253/52, SCO4176/77, SCO4678/79, SCO6236/35, and SCO7615/16) had different phenotypes depending on the culture medium used, their effect was also studied on solid LB, NMMP, and PGA (specific for RED production) media (Figure 3).

The results showed that overexpression of the individual genes *SCO2245* and *SCO2246* reduced the production of ACT (blue color) on LB medium while no effect was detected for the complete SCO2246/45 system. On NMMP, no differences, compared with the control, were observed and, on PGA, only the XRE gene produced a small increase in RED production at 7 days of culture (Figure 3A). For the SCO2253/52 system, the reduction in ACT production was stronger with the overexpression of the gene *SCO2253*, or both genes *SCO2253/52*, than with *SCO2252* on LB. Reduced ACT production was also observed on NMMP when the XRE gene alone or the complete system were overexpressed. On PGA, only the DUF397 gene produced a small increase in RED production at 5 and 7 days of culture. Also, a bald phenotype was observed on LB when gene *SCO2253* was overexpressed (Figure 3B). The analysis of genes *SCO4176/77* on LB revealed that the strongest reduction in ACT production occurred when gene *SCO4176* was overexpressed. Reduced ACT production was observed on NMMP when the XRE or the DUF397 genes were expressed and on PGA only the DUF397 gene produced a small increase in RED production at 5 and 7 days of culture (Figure 3C). The overexpression of system SCO4678/79 led to a drastic reduction in ACT production when individual genes *SCO4678* or *SCO4679* were overexpressed on LB medium. When the gene *SCO4679* (DUF397 gene) or both *SCO4678/79* genes were overexpressed on NMMP, a clear increase in ACT production was observed. However, the overexpression of the *SCO4678* (XRE gene) was associated with a reduction in ACT production on this medium. On PGA, only the expression of both genes led to a small increase in RED production at 5 and 7 days of culture (Figure 3D). The overexpression of gene *SCO6236* or the complete system, SCO6236/35, induced a clear reduction in ACT production when the experiment was done on LB. Reduced ACT production was observed on NMMP when the XRE or DUF397 genes were expressed individually. On PGA, only the DUF397 gene showed a small increase in RED production at 5 and 7 days of culture (Figure 3E). The overexpression of system SCO7615/16 presented a slight decrease in ACT production when both elements were expressed individually or together. A reduction in ACT production was observed on NMMP when the XRE or DUF397 genes were expressed separately and, on PGA, no effect was observed (Figure 3F).

In summary, the overexpression of the six XRE/DUF systems acted as pleiotropic regulators influencing ACT and RED production and/or morphological differentiation. Therefore, in all of the systems analyzed, the overexpression of one gene or both of the genes in the pair provoked a reduction in ACT production on LB medium. Only one of the systems, SCO4678/4679, acted as a positive regulator on NMMP when the DUF gene (*SCO4679*) or the whole system were overexpressed. The rest of the systems provoked, in general, a minor reduction in ACT production on NMMP medium. Conversely, five of the systems had a slightly positive effect on RED production when cultured on PGA.

### Biological activity of the strains that overexpressed the selected XRE/DUF397 systems

**Figure 4:**
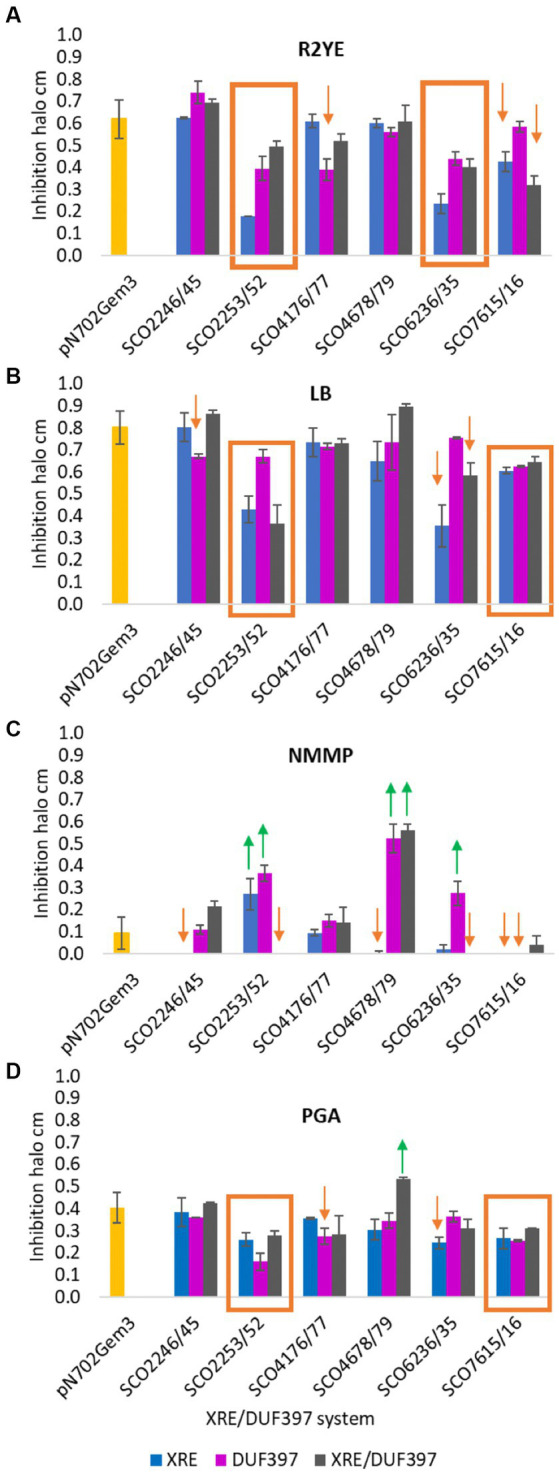
Inhibition halos of antibiograms against *Bacillus subtilis.* Bioactivity over *B. subtilis* of *S. coelicolor* clones overexpressing 6 XRE/DUF397 systems on 4 different culture media. **(A)** R2YE medium; **(B)** LB medium; **(C)** NMMP medium; **(D)** PGA medium. 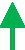 Increased production of bioactive secondary metabolites. 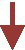 Decreased production of bioactive secondary metabolites. The yellow bar corresponds to the bioactivity of the control *S. coelicolor* M145/pNX702GEM3. The experiment was performed in triplicate.

To complete the phenotypic study of the overexpression of the six selected XRE-DUF397 systems, the production of bioactive secondary metabolites induced under the conditions assayed was analyzed. Antibiograms against *Bacillus subtilis* of each of the constructions were performed. The inhibition halos were an indirect quantification of the antibiotics produced due to the overexpression of the transformants grown on each medium used: R2YE, LB, NMMP, and PGA. As shown in Figure 4, variations in biological activity were observed and were culture media dependent.

In system SCO2245/46, only a decrease in the size of the halo was observed when mutants overexpressing gene *SCO2245* (DUF) were grown on LB or *SCO2246* (XRE) on NMMP (Figures 4B,C).

System SCO2253/52 exerted a negative regulation over antibiotic production on three of the media used: R2YE, LB, and PGA. This reduction was observed with the overexpression of the individual gene and the gene pair (Figures 4A,B,D). However, on NMMP, the individual overexpression of the two elements of this system produced more biological activity than the strain carrying the empty plasmid. However, no biological activity was observed when both genes were overexpressed together (Figure 4C). In order to corroborate the result obtained on LB medium, liquid cultures of the control strain and the strains with the overexpression of the individual gene and the gene pair were made (Supplementary Figure S6). Clearly, the strains that individually overproduced each protein had a strong reduction in ACT production, which was less intense when both genes were expressed. These data are in agreement with the results obtained on solid media as represented in the Supplementary Figure S6. In fact, there was an increase in RED production in liquid (at 4 days) and on solid media (over time) with the strain carrying the plasmid pNX2252.

For the system SCO4176/77, only a small decrease in the inhibitory halo was observed when the overexpressing strains harboring the DUF gene were grown on R2YE and PGA (Figures 4A,D).

For SCO4678/79, the increase in the bioactive products on NMMP was remarkable when the DUF gene or both genes were overexpressed (Figure 4C). This corresponded to the increase in ACT production previously observed on this medium (Figure 3D). In addition, an increase was observed when both genes were overexpressed on PGA.

A general negative effect was observed in the transformants carrying one or two genes of system SCO6235/36 on R2YE medium (Figure 4A), which was also the same on LB and NMMP when XRE or both were overexpressed (Figures 4B,C) and on PGA with the XRE gene (Figure 4D). On the other hand, on NMMP, there was an increase when the DUF gene was the one that was overexpressed (Figure 4C).

Finally, when the SCO7615/16 system was overexpressed either individually or as a pair, a small decrease in antibiotic production was observed (Figures 4A,B,D). This decrease was more evident on NMMP medium, which lacked biological activity when the genes were individually overexpressed (Figure 4C).

### Deletion of the six selected XRE/DUF397 systems causes different effects on antibiotic production and morphological differentiation

To further investigate the role of the six XRE/DUF397 systems selected (SCO2246/45, SCO2253/52, SCO4176/77, SCO4678/79, SCO6236/35, and SCO7615/16) both genes of each system were deleted in *S. coelicolor* M145 using the CRISPR-Cas9 system (Tong et al., 2015; see Materials and methods).

**Figure 5:**
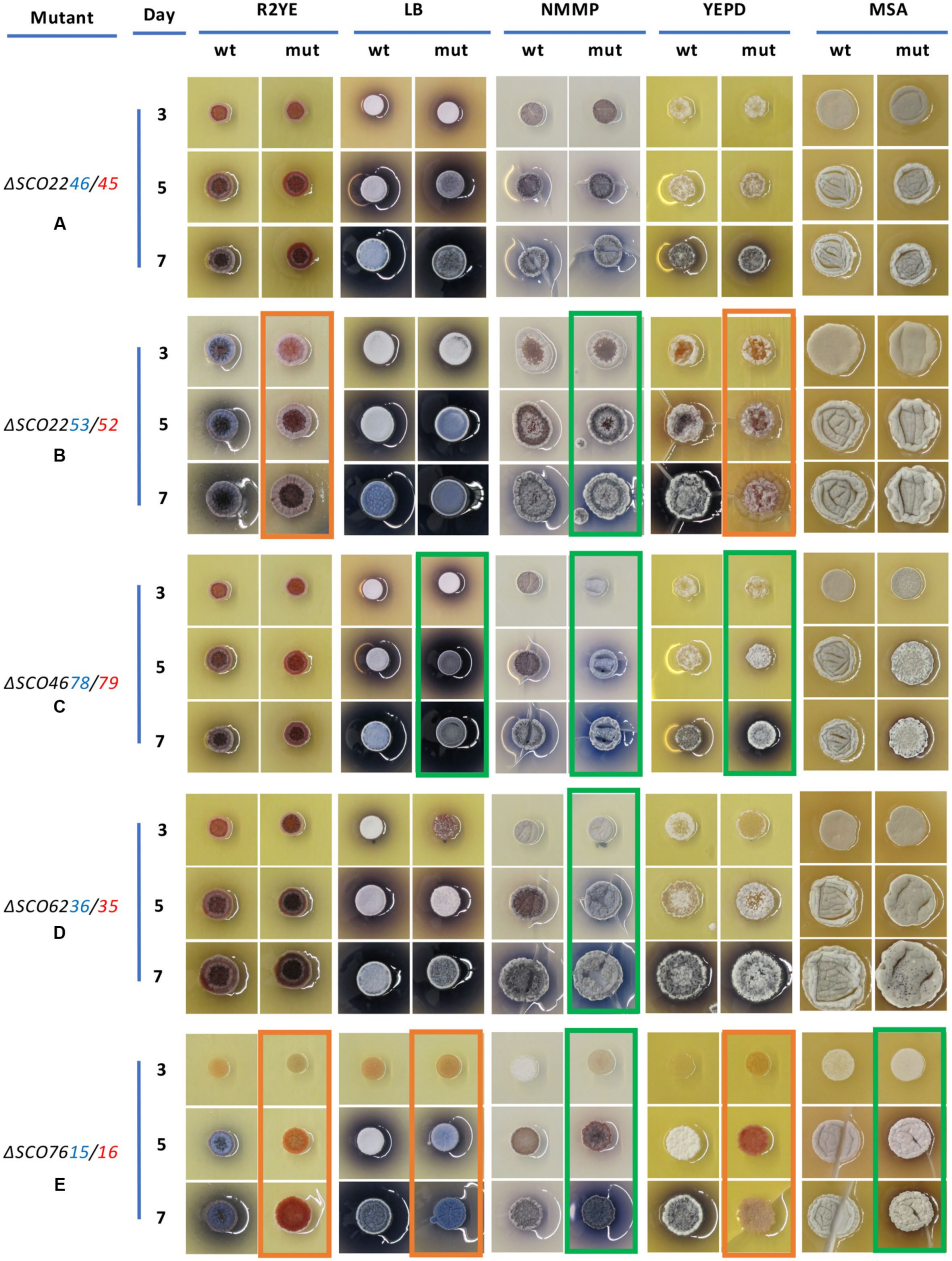
Phenotypes of five XRE/DUF397 system deletion mutants in *S. coelicolor* grown on different solid media. **(A)** Phenotypes of the different mutants (mut) versus the wild type *S. coelicolor* M145 (wt) on the five media assayed: R2YE, LB, NMMP, YEPD, and MSA at 3, 5, and 7 days of culture. **(A)** ∆*SCO2246/45*; **(B)** *∆SCO2253/52*; **(C)** ∆*SCO4176/77*; **(D)** ∆*SCO4678/79*; **(E)** *∆SCO6236/35*. Ο Green, increase in actinorhodin production; Ο Orange, decrease in actinorhodin production.

Deletions were obtained in all systems except for the gene pair *SCO4176/77*, as it was not possible to obtain deletion mutants even after several attempts. The mutants of the other five systems were verified by PCR (Supplementary Figures S7–S11) and their phenotypes were analyzed. Drops containing 5 × 10^5^ spores were inoculated on five different solid media (R2YE, LB, NMMP, YEPD, and MSA), and their development and the production of colored antibiotics were monitored for 7 days (Figure 5).

The *∆SCO2246/45* mutant did not show any relevant phenotypic differences on any of the culture media tested (Figure 5A).

The *∆SCO2253/52* mutant showed a drastic reduction in ACT production when grown on R2YE and YEPD. Also, on YEPD there was a clear delay in the development of aerial mycelium. Conversely, this mutant presented a small increase in ACT production on NMMP (Figure 5B).

On the other hand, the *∆SCO4678/79* mutant did not show any clear phenotypic differences on R2YE, while on LB and NMMP more ACT was produced after 5 days. This increase in ACT production was also observed on YEPD on day 7 (Figure 5C).

Likewise, the *∆SCO6236/35* mutant did not show any clear phenotypic differences on the R2YE, LB, YEPD, or MSA media; however, on NMMP it produced more ACT than the wild type, the difference being much clearer on day 7 (Figure 5D).

Finally, the *∆SCO7615/16* mutant showed a different phenotype on each of the media used. On R2YE and YEPD, a bald phenotype was observed, even after 7 days, with little or no ACT production, as compared to the wild-type strain. On LB medium, lower production of ACT was also observed although the spores produced aerial mycelium. However, on NMMP medium, the mutant produced a greater amount of ACT. Finally, on MSA medium, the mutant produced more ACT and pigments than the wild-type strain (Figure 5E).

**Figure 6:**
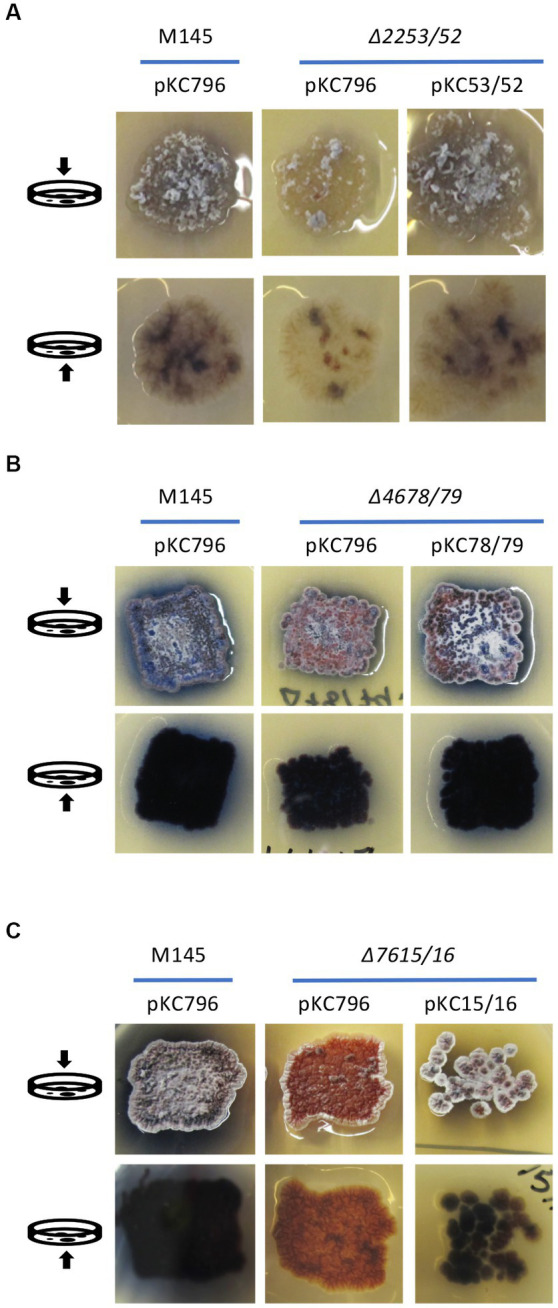
Complementation of *∆SCO2253/52, ∆SCO4678/79,* and *∆SCO7615/16* mutants. **(A)** Complementation of the *∆SCO2253/52* mutant using the integrative plasmid pKC53/52 harboring genes *SCO2253/52* on YEPD. **(B)** Complementation of the *∆SCO4678/79* mutant using the integrative plasmid pKC78/79 harboring genes *SCO4678/79* on R2YE. **(C)** Complementation of the *∆SCO7615/16* mutant using the integrative plasmid pKC15/16 harboring genes *SCO7615/16* on R2YE. The wild-type strain *S. coelicolor* M145 was used as a control and the corresponding mutants were also transformed with the integrative empty plasmid pKC796. The arrows on the left indicates if the image was taken from the top or button of the Petri dishes.

The complementation of these mutant strains was carried out using the integrative plasmid pKC796 harboring the corresponding deleted genes for each case. The phenotypic assays were carried out on those mutants which had the most dramatic differences compared to the wild-type strain; *∆SC2253/52*, *∆SCO4678/79* and *∆SCO7615/16*. The cultures were done on the medium on which was observed the strongest differences between the mutant and wild type strain. As shown in Figure 6, there were complementation of the phenotypes presented by the mutants *∆SCO2253/52* on YEPD medium, and those of the *∆SCO4678/79* and *∆SCO7615/16* on R2YE. Based on these results, it was concluded that the deletion of these genes was responsible for the phenotypes observed.

## Discussion

XRE transcriptional regulators are quite abundant in *Streptomyces* and are known to be associated with pleiotropic functions, among which the regulation of secondary metabolism plays an important role (Santamaría et al., 2018; Tian et al., 2020; Zhu et al., 2020). These regulators have an N-terminal HTH DNA domain and a variable C-terminal region that have been denominated DUF5753 domain. In actinomycetes, a high number of genes encoding XRE regulators are linked to a gene encoding a small protein (63–91 aa) containing a DUF397 domain. It has been proposed that these pairs are homologous to type II toxin/antitoxin, where the DUF397 protein is the toxin and the XRE the antitoxin (Makarova et al., 2009; Doroghazi and Buckley, 2014). *S. coelicolor* has 15 XRE/DUF397 pairs in its genome, of which only the *SCO1979* gene and gene pairs formed by *SCO4543/4542* and *SCO4441/4442* (Scr1/Scr2) have already been shown to play a regulatory role in differentiation and antibiotic production (Aínsa et al., 2010; Santamaría et al., 2018; Zhu et al., 2020).

The role of the XRE/DUF397 proteins in the development of *S. coelicolor* has also previously been shown for the *bldB* (encoding a DUF397 protein) and *bldD* genes (encoding an XRE regulator) (Eccleston et al., 2006). *bld* mutants were originally isolated because they block aerial mycelium formation. *bldB* is one of these genes and encodes an impaired DUF397 protein that is related to 24 other BldB-like proteins, of which 14 have been analyzed in this work. In fact, this *bldB*-like family of genes is unique to filamentous actinomycetes and all of them borrow the high conservation of several amino acids on which, five are important for its function and level of expression (Eccleston et al., 2006). In addition, it has been proposed that the BldB protein may interact with another not identified partner and that this interaction positively affects the development of these bacteria (Eccleston et al., 2006). On the other hand, BldD (a XRE protein) is a master developmental repressor that regulates the transcription of key developmental genes and blocks the initiation of sporulation in *S. coelicolor* (Elliot et al., 2001; den Hengst et al., 2010).

In our study, we have shown that fourteen XRE/DUF397 pairs participate in the regulation of antibiotic production and/or differentiation to a greater or lesser extent. In addition, this regulation could be different in each case and may even be culture media dependent. Here, we have shown that, in *S. coelicolor*, these 14 systems are pleiotropic regulators, generally acting as negative regulators of the development and production of ACT. Therefore, the overexpression of the XRE genes *SCO2253*, *SCO4176*, and *SCO5678* on R2YE lead to bald colonies. This same phenotype was observed in the overexpression of the DUF gene *SCO7177* and in the join expression of pairs *SCO2253/52*, *SCO4176/77*, and *SCO4678/79* on R2YE. However, this effect was not clear when these strains were grown on other types of media such as LB and NMMP, suggesting that the composition of the media had a strong effect on development. Concerning ACT, less antibiotic production was observed on R2YE when the DUF genes *SCO1978*, *SCO2245*, *SCO6236*, and *SCO7615* or the pair *SCO1979/78* were overexpressed. However, this analysis of the overexpression of the 14 putative toxins (DUF397 proteins) showed no clear toxic effect. Thus, the number of *S. coelicolor* colonies obtained for the transformants was similar with the DNAs containing each of the individual genes and with the DNAs containing both genes. Moreover, all the colonies obtained grew normally on the four solid media used (R2YE, LB, NMMP, and PGA) (Figures 2, 3).

Recently, it has been reported that the system SCO4678/79 is included in a 17 kb-long region of the chromosome that was overrepresented in the analysis of the components included in the membrane vesicles produced by *S. coelicolor*. Interestingly, this region of the chromosome was previously denominated “the supercoiling-hypersensitive cluster (SHC).” This SCH region includes 34 genes (from *SCO4667* to *SCO4700*), of which 26 of them (that includes the pair *SCO4678/79*) are supercoiling sensitive and poorly transcribed under standard culture conditions. However, they are strongly upregulated under both topoisomerase I-downregulation and topoisomerase I-upregulation conditions (Szafran et al., 2019; Faddetta et al., 2022).

Further studies on these gene pairs (*XRE/DUF397*) may be necessary to complete their cascade of regulation; in particular those pairs whose mutation present more drastic effects such as the gene pair *SCO7615/16*.

## Data availability statement

The original contributions presented in the study are included in the article/ Supplementary material, further inquiries can be directed to the corresponding authors.

## Author contributions

CR and AM-C conducted the experiments. RS and MD designed the experiments and wrote the manuscript. All authors have read and approved the final manuscript.

## Funding

This work has been funded by project PID2019-107716RB-I00 (Spanish Ministry of Science and Innovation/State Research Agency/10.13039/501100011033). In addition, the Institute of Biology and Functional Genomics (IBFG) has received funding through the program “Escalera de Excelencia” of the Regional Government of Castile and Leon (ref.: CLU-2017-03) and co-financed by the P.O. FEDER of Castile and León 14–20, and the Internationalization Project “CL-EI-2021-08-IBFG Unit of Excellence” of the Spanish National Research Council (CSIC), funded by the Regional Government of Castile and Leon and co-financed by the European Regional Development Fund (ERDF “Europe drives our growth).” CR was financially supported by “Minciencias” No. 860 of 2019 and by a mobility grant from Santander Bank and the University of Salamanca.

## Conflict of interest

The authors declare that the research was conducted in the absence of any commercial or financial relationships that could be construed as a potential conflict of interest.

## Publisher’s note

All claims expressed in this article are solely those of the authors and do not necessarily represent those of their affiliated organizations, or those of the publisher, the editors and the reviewers. Any product that may be evaluated in this article, or claim that may be made by its manufacturer, is not guaranteed or endorsed by the publisher.

## Footnotes

^1^ https://bioinfo-mml.sjtu.edu.cn/TADB2/index.php

^2^ http://streptomyces.org.uk/actinoblast/
