# Supplementary material for: Role of fourteen XRE-DUF397 pairs from Streptomyces coelicolor as regulators of antibiotic production and differentiation. New players in a complex regulatory network
Source: Front Microbiol. 2023 Jul 10;14:1217350. doi: 10.3389/fmicb.2023.1217350 (PMC10364602; doi:10.3389/fmicb.2023.1217350)
Supplement: Supplementary file 1 [file Data_Sheet_1.PDF]

Supplementary Material

Role of Fourteen XRE-DUF397 Pairs from *Streptomyces coelicolor* as Regulators of Antibiotic Production and Differentiation. New Players in a Complex Regulatory Network.

Carolina Riascos Cuero, Ana Martínez-Carrasco, Margarita Díaz* and Ramón I. Santamaría*

1.- Instituto de Biología Funcional y Genómica (IBFG)/Departamento de Microbiología y Genética. Consejo Superior de Investigaciones Científicas (CSIC)/Universidad de Salamanca (USAL), C/ Zacarías González, nº 2, 37007-Salamanca, Spain

E-mail addresses:

CR: carolinariascos@usal.es Tel: +34-923-294889.

AMC: amcgar@idaea.csic.es Tel:+34637247505

*MD: mardi@usal.es Tel: +34-923-294400 Ext: 5418

*RIS: santa@usal.es Tel: +34-923-294899.

***Co-Address correspondence**

Supplementary Table 1: Oligonucleotides used in this work

Primer	Sequences (5'- 3')	Application
CR-007	TTTTTTCATATGAACCGCATCAAGCCC	Forward primer for amplifying *SCO1978*. NdeI site is underlined.
CR-032	TATGATATCTCAGGACAGAAGGAAGTCCGCCTC	Reverse primer for amplifying *SCO1978*. EcoRV site is underlined.
CR-009	TTTTTTCATATGGTGAGCGAGCCGCGGTCC	Forward primer for amplifying *SCO1979*. NdeI site is underlined.
CR-031	TATGATATCTCACAGCTCCTTGCGGAGATCCCGG	Reverse primer for amplifying *SCO1979*. EcoRV site is underlined.
CR-001	TTTTTTCATATGTCGGTGAACGGCGAGGCG	Forward for amplifying SCO2246. NdeI site is underlined
CR-002	TTTTTTCTCGAGCCGCAGTTCGAGGATGCG	Reverse for amplifying SCO2246. XhoI site is underlined
CR-003	TTTTTTCATATGACCGGTAAGGACACACCCGTACAC	Forward for amplifying SCO2245. NdeI site is underlined
CR-004	TTTTTTCTCGAGTTCCGCGAGCGTCCCGGC	Reverse for amplifying SCO2245. XhoI site is underlined
CR-033	TTTTTTCATATGAACAGCGTCACCTCAGC	Forward for amplifying SCO2252. NdeI site is underlined
CR-034	TTTTTTCTCGAGAACCCGAGGACTGAGCG	Reverse for amplifying SCO2252. XhoI site is underlined
CR-035	TTTTTTCATATGTCGGTGAACGGCGAGG	Forward for amplifying SCO2253. NdeI site is underlined
CR-036	TTTTTTCTCGAGTGACTCTCCAAGCATCTGCTCG	Reverse for amplifying SCO2253. XhoI site is underlined
CR-085	TTTTTTCATATGGTGGCCAACATCCAGTCCC	Forward primer for amplifying *SCO2381*. NdeI site is underlined.
CR-086	TTTTTTCTCGAGACATTGCTCAGTGCTGTGC	Reverse primer for amplifying *SCO2381*. XhoI site is underlined.
CR-087	TTTTTTCATATGCGGTCCCGCACAG	Forward primer for amplifying *SCO2382*. NdeI site is underlined.
CR-088	TTTTTTCTCGAGACGCAGGCCGTCGAC	Reverse primer for amplifying *SCO2382*. XhoI site is underlined.
CR-038	TTTTTTCATATGGCCAATGGTTCACGGCAGG	Forward primer for amplifying *SCO2513*. NdeI site is underlined.
CR-039	TTTTTTCTCGAGGGTCGTACTCGCGCATCGTCTG	Reverse primer for amplifying *SCO2513*. XhoI site is underlined.
CR-040	TTTTTTCATATGCGCGAGTACGACCTGACG	Forward primer for amplifying *SCO2514*. NdeI site is underlined.
CR-041	TTTTTTCTCGAGAACACCCGCTCCGCGG	Reverse primer for amplifying *SCO2514*. XhoI site is underlined.
CR-022	TTTTTTCATATGGTGCCGCAGCGGAGGG	Forward for amplifying SCO4176. NdeI site is underlined

CR-027	TTTTTTCTCGAGGGTCGATGGCTCGCATGGC	Reverse for amplifying SCO4176. XhoI site is underlined
CR-028	TTTTCATATGTCTTGGCGCAGGAGCAGCTACAGCAACTC	Forward for amplifying SCO4177. NdeI site is underlined
CR-029	TTTTTTCTCGAGCGCGGCCCGCCCCGG	Reverse foramplifying SCO4177. XhoI site is underlined
CR-044	TTTTTTCATATGGAGACCACCCCTGAGTACG	Forward primer for amplifying *SCO4300*. NdeI site is underlined.
CR-045	TTTTTTCTCGAGCCGTCCACCTGGGCCTGA	Reverse primer for amplifying *SCO4300*. XhoI site is underlined.
CR-042	TTTTTTCATATGCCAGGACCGAAAGACCTCG	Forward primer for amplifying *SCO4301*. NdeI site is underlined.
CR-043	TTTTTTCTCGAGGGGGTGGTCTCCATGCGC	Reverse primer for amplifying *SCO4301*. XhoI site is underlined.
CR-064	TTTTTTCATATGGTGACCGTACGCCCCC	Forward primer for amplifying *SCO4542*. NdeI site is underlined.
CR-065	TTTTTTCTCGAGGACAGTGGACCTCTTGAGGCTC	Reverse primer for amplifying *SCO4542*. XhoI site is underlined.
CR-062	TTTTTTCATATGGTGGCGCCAAGGAGTCG	Forward primer for amplifying *SCO4543*. NdeI site is underlined.
CR-063	TTTTTTCTCGAGCAATTCCTTCGCCAGGTGGT	Reverse primer for amplifying *SCO4543*. XhoI site is underlined.
CR-066	TTTTTTCATATGGCGGTCGAGACGGAC	Forward for amplifying SCO4678. NdeI site is underlined
CR-067	TTTTTTCTCGAGTGTGTCTCCCAGCAGTTTTTCG	Reverse foramplifying SCO4678. XhoI site is underlined
CR-068	TTTTTTCATATGATCCGCGAGACCGAGC	Forward for amplifying SCO4679. NdeI site is underlined
CR-069	TTTTTTCTCGAGGCGCCGCGACGCGTA	Reverse for amplifying SCO4679. XhoI site is underlined
CR-052	TTTTTTCATATGATCCGCACGTCCTCGTCCA	Forward primer for amplifying *SCO5124*. NdeI site is underlined.
CR-053	TTTTTTCTCGAGACTTCTGACCGTGCCGGC	Reverse primer for amplifying *SCO5124*. XhoI site is underlined.
CR-050	TTTTTTCATATGTCGGTGGACGCCGG	Forward primer for amplifying *SCO5125*. NdeI site is underlined.
CR-051	TTTTTTCTCGAGTGTCTCCCCCAGCAGTTGCTCG	Reverse primer for amplifying *SCO5125*. XhoI site is underlined.
CR-083	TTTTTTCATATGAGCAGCACCGCACTTCG	Forward primer for amplifying *SCO6128*. NdeI site is underlined.
CR-084	TTTTTTCTCGAGCCGTGCCCCGAGGAACGC	Reverse primer for amplifying *SCO6128*. XhoI site is underlined.
CR-081	TTTTTTCATATGGTGGTCATGATGGAACAGTGCGC	Forward primer for amplifying *SCO6129*. NdeI site is underlined.

CR 082	TTTTTTCTCGAGTTGCTCTCCTAGGAGACGGTCCAG	Reverse primer for amplifying *SCO6129*. XhoI site is underlined.
CR-058	TTTTTTCATATGAACACGACGGAACTGGCC	Forward for amplifying SCO6235. NdeI site is underlined
CR-059	TTTTTTCTCGAGGCGGTCGGTCGTGTACGT	Reverse for amplifying SCO6235. XhoI site is underlined
CR-060	TTTTTTCATATGGGCCCGTTGAGTGGG	Forward for amplifying SCO6236. NdeI site is underlined
CR-061	TTTTTTCTCGAGTGAGGCTCCTCGCATCCGC	Reverse for amplifying SCO6236. XhoI site is underlined
CR-011	TTTTTTCATATGGTGGGCGACGAGGTTCAG	Forward primer for amplifying *SCO6629*. NdeI site is underlined.
CR-012	TTTTTTCTCGAGTCTCTCTCCCAACAGCTTCTCG	Reverse primer for amplifying *SCO6629*. XhoI site is underlined.
CR-013	TTTTTTCATATGAACGCTGAAGCAAAGCGC	Forward primer for amplifying *SCO6630*. NdeI site is underlined.
CR-014	TTTTTTCTCGAGGCGTGCCGCGAACTCCAC	Reverse primer for amplifying *SCO6630*. XhoI site is underlined.
CR-077	TTTTTTCATATGGTGAGTGAAGCTCGGTCCGG	Forward for amplifying SCO7615. NdeI site is underlined
CR-078	TTTTTTCTCGAGTGAGTACTCCTTGCGCATGCGG	Reverse for amplifying SCO7615. XhoI site is underlined
CR-079	TTTTTTCATATGAGCACCACCGACGTCC	Forward for amplifying SCO7616. NdeI site is underlined
CR-080	TTTTTTCTCGAGTTCCGTTCCCTTCCTGCGGC	Reverse for amplifying SCO7616. XhoI site is underlined
CR-005	CACTCCCCTTGTTCCACAGC	Forward primer for amplifying inserts in the NdeI and XhoI enzyme cleavage site.
CR-006	CGACTCTAGCTAGAGGTACGGGC	Reverse primer for amplifying inserts in the NdeI and XhoI enzyme cleavage site.
CR-030	CATGCCATGG***GCGGTCGAGTCGCTTTCGTA***GTTTTAGAGCTAGAAATAGC	Forward primer for sgRNA *SCO2246* cassette construction. Specific *SCO2246* sequence in bold italic. NcoI site is underlined.
CR-017	TCCTGGACAAAACTGAGTTC	Forward primer for verifying pCRISPR-Cas9-sg46 plasmids.
CR-070	TTTTTTAGGCCTTCTAGACCACGGGGTAGGGCTGC	Forward primer for building of *SCO2245* upstream homologous template for pCRISPR-Cas9. StuI and XbaI sites is underlined.
CR-071	GTCCGAGTGACACAGTGGCCCTCCGCCAGTGGCATACCGG	Reverse primer for building of *SCO2245* upstream homologous template for pCRISPR-Cas9.
CR-072	CCGGTATGCCACTGGCGGAGGGCCACTGTGTCACTCGGAC	Forward primer for building of *SCO2246* downstream homologous template for pCRISPR-Cas9.

CR-073	TTTTTTAGGCCTTCTAGAGACCCTGCGACCGAGTGATG	Reverse primer for building of *SCO2246* downstream homologous template for pCRISPR-Cas9. StuI and XbaI site is underlined.
CR-074	GCAACGCATTCGGCACG	Reverse primer for verifying the centre of pCRISPR-Cas9-46/45 homology templates.
CR-075	CCAGGTCCGCCGCGACGA	Forward primer for verifying the centre of pCRISPR-Cas9-46/45 homology templates.
CR-092	CATGCCATGG***TCGTGCCGCAACCATGCTCT***GTTTTAGAGCTAGAAATAGC	Forward primer for sgRNA *SCO2253* cassette construction. Specific *SCO2253* sequence in bold italic. NcoI site is underlined.
CR-093	TCGTGCCGCAACCATGCTCT	Forward primer for verifying pCRISPR-Cas9-sg53 plasmids.
CR-094	TTTTTTAGGCCTTCTAGATTCGTTCCGATGCTCGACGA	Forward primer for building of *SCO2252* upstream homologous template for pCRISPR-Cas9. StuI and XbaI sites is underlined.
CR-095	GTCGCGATACGACGCGTACGCCTTCGAGGCCACGAAGAGC	Reverse primer for building of *SCO2252* upstream homologous template for pCRISPR-Cas9.
CR-096	GCTCTTCGTGGCCTCGAAGGCGTACGCGTCGTATCGCGAC	Forward primer for building of *SCO2253* downstream homologous template for pCRISPR-Cas9.
CR-097	TTTTTTAGGCCTTCTAGACCTCATGGCGCTGTTCGT	Reverse primer for building of *SCO2253* downstream homologous template for pCRISPR-Cas9. StuI and XbaI site is underlined.
CR-104	CGCCACCACCCGGAGTAC	Forward primer for verifying the centre of pCRISPR-Cas9-53/52 homology templates.
CR-105	GGTCATGGAGAAGGCCGC	Reverse primer for verifying the centre of pCRISPR-Cas9-53/52 homology templates.
CR-116	CATGCCATGG***TCGGACAGAATGACGCGAAA***GTTTTAGAGCTAGAAATAGC	Forward primer for sgRNA *SCO4176* cassette construction. Specific *SCO4176* sequence in bold italic. NcoI site is underlined.
CR-117	TCGGACAGAATGACGCGAAA	Forward primer for verifying pCRISPR-Cas9-sg76 plasmids.
CR-118	TTTTTTTCTAGACACTGGGTGCACAGCGTCAC	Forward primer for building of *SCO4176* upstream homologous template for pCRISPR-Cas9. XbaI sites is underlined.
CR-119	GCATAAACAGGGCCGGCCTCCCTCCCGGCTGTGGAATTTG	Reverse primer for building of *SCO4176* upstream homologous template for pCRISPR-Cas9.
CR-120	CAAATTCCACAGCCGGGAGGGAGGCCGGCCCTGTTTATGC	Forward primer for building of *SCO4177* downstream homologous template for pCRISPR-Cas9.
CR-121	TTTTTTTCTAGAACCAGGTGGATGTGGTGGTG	Reverse primer for building of *SCO4177* downstream

		homologous template for pCRISPR-Cas9. XbaI site is underlined.
CR-122	CAACTCAAGCGGTGGCAAC	Forward primer for verifying the centre of pCRISPC-Cas9-76/77 homology templates.
CR-123	GAAGCCGTGCTCCGACTC	Reverse primer for verifying the centre of pCRISPR-Cas9-76/77 homology templates.
CR-098	CATGCCATGG***ACGGCCGGTTGGCGGCACAA***GTTTTAGAGCTAGAAATAGC	Forward primer for sgRNA *SCO4678* cassette construction. Specific *SCO4678* sequence in bold italic. NcoI site is underlined.
CR-099	ACGGCCGGTTGGCGGCACAA	Forward primer for verifying pCRISPR-Cas9-sg78 plasmids.
CR-100	TTTTTTAGGCCTGAGCGACTTGATGTCGGCGC	Forward primer for building of *SCO4678* upstream homologous template for pCRISPR-Cas9. StuI site is underlined.
CR-101	GTCGGCACGCAGGAGGTCAGACAGTCACCGTACGTACCCG	Reverse primer for building of *SCO4678* upstream homologous template for pCRISPR-Cas9.
CR-102	CGGGTACGTACGGTGACTGTCTGACCTCCTGCGTGCCGAC	Forward primer for building of *SCO4679* downstream homologous template for pCRISPR-Cas9.
CR-103	TTTTTTTCTAGATGGCGTAGTTCACGACGACG	Reverse primer for building of *SCO4679* downstream homologous template for pCRISPR-Cas9. XbaI site is underlined.
CR-106	CGATGGTCCGTACGCTGC	Reverse primer for verifying the centre of pCRISPR-Cas9-78/79 homology templates.
CR-107	CGTTGTTGGCCAGCTCGG	Forward primer for verifying the centre of pCRISPR-Cas9-78/79 homology templates.
CR-108	CATGCCATGG***CGTCGCCCAATGGTGGCAGT***GTTTTAGAGCTAGAAATAGC	Forward primer for sgRNA *SCO6236* cassette construction. Specific *SCO6236* sequence in bold italic. NcoI site is underlined.
CR-109	CGTCGCCCAATGGTGGCAGT	Forward primer for verifying pCRISPR-Cas9-sg36 plasmids.
CR-110	TTTTTTAGGCCTCAACCACGCCTACACCTGGT	Forward primer for building of *SCO6235* upstream homologous template for pCRISPR-Cas9. StuI site is underlined.
CR-111	GCGGACGGTGGACAGTAAGATCGGTCGGGTCAGTGGTCCG	Reverse primer for building of *SCO6235* upstream homologous template for pCRISPR-Cas9.
CR-112	CGGACCACTGACCCGACCGATCTTACTGTCCACCGTCCGC	Forward primer for building of *SCO6236* downstream homologous template for pCRISPR-Cas9.
CR-113	TTTTTTTCTAGACGGATGTCCTCACCGTGCAC	Reverse primer for building of *SCO6236* downstream homologous template for

		pCRISPR-Cas9. XbaI site is underlined.
CR-114	GTGTCGGACAGCGCCGAG	Reverse primer for verifying the centre of pCRISPR-Cas9-36/35 homology templates.
CR-115	CCCGAACTCGCTGACGAG	Forward primer for verifying the centre of pCRISPR-Cas9-36/35 homology templates.
CR-124	CATGCCATGG***AGCAGCCCTGTGACGTAGTG***GTTTTAGAGCTAGAAATAGC	Forward primer for sgRNA *SCO7615* cassette construction. Specific *SCO7615* sequence in bold italic. NcoI site is underlined.
CR-125	AGCAGCCCTGTGACGTAGTG	Forward primer for verifying pCRISPR-Cas9-sg15 plasmids.
CR-126	TTTTTTAGGCCTTCCCGCGATCCCCAGGTGAC	Forward primer for building of *SCO7615* upstream homologous template for pCRISPR-Cas9. StuI site is underlined.
CR-127	GTGAAGATGCCCGGGTAGGCGGCGCCATTGTGACAGCGAC	Reverse primer for building of *SCO7615* upstream homologous template for pCRISPR-Cas9.
CR-128	GTCGCTGTCACAATGGCGCCGCCTACCCGGGCATCTTCAC	Forward primer for building of *SCO7616* downstream homologous template for pCRISPR-Cas9.
CR-129	TTTTTTTCTAGACTGCTGAGCGCCGTCTGGTC	Reverse primer for building of *SCO7616* downstream homologous template for pCRISPR-Cas9. XbaI site is underlined.
CR-130	CGGGGTGCTGCGCATGAG	Reverse primer for verifying the centre of pCRISPR-Cas9-15/16 homology templates.
CR-131	GCGGAGCGAGGGAAGGTC	Forward primer for verifying the centre of pCRISPR-Cas9-15/16 homology templates.
CR-089	CACAGGGCTCTCCCGTGCC	Reverse external primer for verifying Δ*46/45* genomic deletion.
CR-141	GGCCATGCGGCACTGTTG	Forward external primer for verifying Δ*53/52* genomic deletion.
CR-135	ACGCCCAGCCTACGACTC	Reverse external primer for verifying Δ*76/77* genomic deletion.
CR-137	GATACGCGTGGGCCCTTC	Forward external primer for verifying Δ*78/79* genomic deletion.
CR-138	CTGTGGTTCACCCCGGCG	Reverse internal primer for verifying Δ*78/79* genomic deletion.
CR-134	TCGATGCGCTTCTTGGCG	Forward external primer for verifying Δ*36/35* genomic deletion.
CR-139	GGTCCCGAGAGCGGCCAC	Forward external primer for verifying Δ*15/16* genomic deletion.
CR-140	CGAGTGGCACGGTGCATG	Reverse internal primer for verifying Δ*15/16* genomic deletion.
SAM-056	TCCACACGTGGCACCGCGAT	Primer for sequencing of sgRNA cassettes.

SAM-057	AGAGCATCACCGGCCTGTAC	Primer for sequencing of homology templates inserted in XbaI site. pCRISPR-Cas9 plasmid.
SAM-058	TAACGTCTGGAAAGACGACA	Primer for sequencing of homology templates inserted in XbaI site. pCRISPR-Cas9 plasmid.
CR-150	TTTTTTAGATCTGTCTCTCGCCTCCTTCCG	Forward primer for amplify *SCO2246/45* genes under its own promoter. BglII site is underlined.
CR-151	TTTTTTTCTAGACGTGCGTCCGAGGTAGAC	Reverse primer for amplify *SCO2246/45* genes under its own promoter. XbaI site is underlined.
CR-146	TTTTTTAGATCTCGGATTCGGGGCCACCTC	Forward primer for amplify *SCO2253/52* genes under its own promoter. BglII site is underlined.
CR-147	TTTTTTTCTAGAAGTCCTCATCGCGGCAAC	Reverse primer for amplify *SCO2253/52* genes under its own promoter. XbaI site is underlined.
CR-144	TTTTTTAGATCTCAGCCCCCAGGAACGCAG	Forward primer for amplify *SCO4678/79* genes under its own promoter. BglII site is underlined.
CR-145	TTTTTTCTAGACTGTGGTTCACCCCGGCG	Reverse primer for amplify *SCO4678/79* genes under its own promoter. XbaI site is underlined.
CR-142	TTTTTTAGATCTGACAGCGCCGAGCCGTAC	Forward primer for amplify *SCO6236/35* genes under its own promoter. BglII site is underlined.
CR-143	TTTTTTTCTAGAGGCGGGGCGTTTCCAGTG	Reverse primer for amplify *SCO6236/35* genes under its own promoter. XbaI site is underlined.
CR-148	TTTTTTAGATCTGCGAGGGAAGGTCGAACC	Forward primer for amplify *SCO7615/16* genes under its own promoter. BglII site is underlined.
CR-149	TTTTTTTCTAGACCGGGAAGTGGTCCTTGC	Reverse primer for amplify *SCO7615/16* genes under its own promoter. XbaI site is underlined.
CR-152	GTCGTGCTATCCGTAAGG	Primer for sequencing of homology templates inserted in BglII site. pKC796 plasmid.
CR-153	CAGGAAACAGCTATGACC	Primer for sequencing of homology templates inserted in XbaI site. pKC796 plasmid.

Supplementary table 2: plasmids constructed and used in this work

Plasmid	Characters	Reference
pXHis1	pBluescript SK derivative. Ampicillin resistance. The xysA promoter from S. halstedii controls xys1D expression.	(Adham et al., 2001)
pX1978	Derived from pXHis Includes the *SCO1978* gene	This work
pX1979	Derived from pXHis Includes the *SCO1979* gene	
pX1978/79	Derived from pXHis Includes the *SCO1978-SCO1979* genes	
pX2245	Derived from pXHis Includes the *SCO2245* gene	
pX2246	Derived from pXHis Includes the *SCO2246* gene	
pX2246/45	Derived from pXHis Includes the *SCO2246-SCO2245* genes	
pX2252	Derived from pXHis Includes the *SCO2252* gene	
pX2253	Derived from pXHis Includes the *SCO2253* gene	
pX2253/52	Derived from pXHis Includes the *SCO2252-SCO2253* genes	
pX2381	Derived from pXHis Includes the *SCO2381* gene	
pX2382	Derived from pXHis Includes the *SCO2382* gene	
pX2381/82	Derived from pXHis Includes the *SCO2381-SCO2382* genes	
pX2513	Derived from pXHis Includes the *SCO2513* gene	
pX2514	Derived from pXHis Includes the *SCO2514* gene	
pX2513/14	Derived from pXHis Includes the *SCO2513-SCO2514* genes	
pX4176	Derived from pXHis Includes the *SCO4176* gene	
pX4177	Derived from pXHis Includes the *SCO4177* gene	
pX4176/77	Derived from pXHis Includes the *SCO4176-SCO4177* genes	
pX4300	Derived from pXHis Includes the *SCO4300* gene	
pX4301	Derived from pXHis Includes the *SCO4301* gene	
pX4301/00	Derived from pXHis Includes the *SCO4301-SCO4300* genes	

pX4542	Derived from pXHis Includes the *SCO4542* gene	
pX4543	Derived from pXHis Includes the *SCO4543* gene	
pX4543/42	Derived from pXHis Includes the *SCO4543-SCO4542* genes	
pX4678	Derived from pXHis Includes the *SCO4678* gene	
pX4679	Derived from pXHis Includes the *SCO4679* gene	
pX4678/79	Derived from pXHis Includes the *SCO4678-SCO4679* genes	
pX5124	Derived from pXHis Includes the *SCO5124* gene	
pX5125	Derived from pXHis Includes the *SCO5125* gene	
pX5125/24	Derived from pXHis Includes the *SCO5125-SCO5124* genes	
pX6128	Derived from pXHis Includes the *SCO6128* gene	
pX6129	Derived from pXHis Includes the *SCO6129* gene	
pX6129/28	Derived from pXHis Includes the *SCO6129-SCO6128* genes	
pX6235	Derived from pXHis Includes the *SCO6235* gene	
pX6236	Derived from pXHis Includes the *SCO6236* gene	
pX6236/35	Derived from pXHis Includes the *SCO6236-SCO6235* genes	
pX6629	Derived from pXHis Includes the *SCO6629* gene.	
pX6630	Derived from pXHis Includes the *SCO6630* gene.	
pX6629/30	Derived from pXHis Includes the *SCO6629-SCO6630* genes.	
pX7615	Derived from pXHis Includes the *SCO7615* gene	
pX7616	Derived from pXHis Includes the *SCO7616* gene	
pX7615/16	Derived from pXHis Includes the *SCO7615-SCO7616* genes	
pNX4	Derived from pN702GEM3. Expression of xylanase Xys1 truncated with xysA promoter from Streptomyces halstedii.	(Díaz et al., 2005)
pNX1978	Derived from pNX4 Includes the *SCO1978* gene	This work
pNX1979	Derived from pNX4 Includes the *SCO1979* gene	

pNX1979/78	Derived from pNX4 Includes the *SCO1979-SCO1978* genes	
pNX2245	Derived from pNX4 Includes the *SCO2245* gene	
pNX2246	Derived from pNX4 Includes the *SCO2246* gene	
pNX2246/45	Derived from pNX4 Includes the *SCO2246-SCO2245* genes	
pNX2252	Derived from pNX4 Includes the *SCO2252* gene	
pNX2253	Derived from pNX4 Includes the *SCO2253* gene	
pNX2253/52	Derived from pNX4 Includes the *SCO2253-SCO2252* genes	
pNX2381	Derived from pNX4 Includes the *SCO2381* gene	
pNX2382	Derived from pNX4 Includes the *SCO2382* gene	
pNX2381/82	Derived from pNX4 Includes the *SCO2381-SCO2382* genes	
pNX2513	Derived from pNX4 Includes the *SCO2513* gene	
pNX2514	Derived from pNX4 Includes the *SCO2514* gene	
pNX2513/14	Derived from pNX4 Includes the *SCO2513-SCO2514* genes	
pNX4176	Derived from pNX4 Includes the *SCO4176* gene	
pNX4177	Derived from pNX4 Includes the *SCO4177* gene	
pNX4176/77	Derived from pNX4 Includes the *SCO4176-SCO4177* genes	
pNX4300	Derived from pNX4 Includes the *SCO4300* gene	
pNX4301	Derived from pNX4 Includes the *SCO4301* gene	
pNX4301/00	Derived from pNX4 Includes the *SCO4301-SCO4300* genes	
pNX4542	Derived from pNX4 Includes the *SCO4542* gene	
pNX4543	Derived from pNX4 Includes the *SCO4543* gene	
pNX4543/42	Derived from pNX4 Includes the *SCO4543-SCO4542* genes	
pNX4678	Derived from pNX4 Includes the SCO4678 gene	
pNX4679	Derived from pNX4 Includes the SCO4679 gene	

pNX4678/79	Derived from pNX4 Includes the SCO4678-SCO4679 genes	
pNX5124	Derived from pNX4 Includes the *SCO5124* gene	
pNX5125	Derived from pNX4 Includes the *SCO5125* gene	
pNX5125/24	Derived from pNX4 Includes the *SCO5125-SCO5124* genes	
pNX6128	Derived from pNX4 Includes the *SCO6128* gene	
pNX6129	Derived from pNX4 Includes the *SCO629* gene	
pNX6129/28	Derived from pNX4 Includes the *SCO6129-SCO6128* genes	
pNX6235	Derived from pNX4 Includes the *SCO6235* gene	
pNX6236	Derived from pNX4 Includes the *SCO6236* gene	
pNX6236/35	Derived from pNX4 Includes the *SCO6236-SCO6235* genes	
pNX6629	Derived from pNX4 Includes the *SCO6629* gene.	
pNX6630	Derived from pNX4 Includes the *SCO6630* gene.	
pNX6629/30	Derived from pNX4 Includes the *SCO6629-SCO6630* genes.	
pNX7615	Derived from pNX4 Includes the *SCO7615* gene	
pNX7616	Derived from pNX4 Includes the *SCO7616* gene	
pNX7615/16	Derived from pNX4 Includes the *SCO7615-SCO7616* genes	
pCRISPR-Cas9	Bifunctional plasmid *Escherichia* – Streptomyces. This plasmid includes cas9 gene from *Streptococcus pyogenes* under tipA promoter, and sgRNA cassette under ermE promoter. Thermosensitive. Resistances: Apramycin, Thiostrepton.	(Tong et al., 2015)
pCRISPR-Cas9-sg2246	pCRISPR-Cas9 derivative. sgRNA targeted to SCO2246/45 system.	This work
pCRISPR-Cas9-2246/45	pCRISPR-Cas9-sgB derivative. This plasmid includes the homologous recombination template for adjacent sequences to *SCO2246/45* system genes.	
pCRISPR-Cas9-sg2253	pCRISPR-Cas9 derivative. sgRNA targeted to SCO2253/52 system.	

pCRISPR-Cas9-2253/52	pCRISPR-Cas9-sgB derivative. This plasmid includes the homologous recombination template for adjacent sequences to *SCO2253/52* system genes.	
pCRISPR-Cas9-sg4678	pCRISPR-Cas9 derivative. sgRNA targeted to SCO4678/79 system.	
pCRISPR-Cas9-4678/79	pCRISPR-Cas9-sgB derivative. This plasmid includes the homologous recombination template for adjacent sequences to *SCO4678/79* system genes.	
pCRISPR-Cas9-sg6236	pCRISPR-Cas9 derivative. sgRNA targeted to SCO6236/35 system.	
pCRISPR-Cas9-6236/35	pCRISPR-Cas9-sgB derivative. This plasmid includes the homologous recombination template for adjacent sequences to *SCO6236/35* system genes.	
pCRISPR-Cas9-sg7615	pCRISPR-Cas9 derivative. sgRNA targeted to SCO7615/16 system.	
pCRISPR-Cas9-7615/16	pCRISPR-Cas9-sgB derivative. This plasmid includes the homologous recombination template for adjacent sequences to *SCO7615/16* system genes.	
pUZ8002	Plasmid to conjugative transfer (operon tra). Resistances: Neomycin.	(Paget et al., 1999)
pKC796	Bifunctional plasmid *Escherichia – Streptomyces.* Integrative (contains phage φC31 integrase). Resistances: Apramycin.	(Kuhstoss et al., 1991)
pKC2246/45	pKC796 derivative. This plasmid includes *SCO2246/45* genes under its own promoter	This work
pKC2253/52	pKC796 derivative. This plasmid includes *SCO2253/52* genes under its own promoter	
pKC2278/79	pKC796 derivative. This plasmid includes *SCO4678/79* genes under its own promoter.	
pKC2236/35	pKC796 derivative. This plasmid includes *SCO6236/35* genes under its own promoter.	
pKC2215/16	pKC796 derivative. This plasmid includes *SCO7615/16* genes under its own promoter.	

Adham, S.A., Campelo, A.B., Ramos, A., and Gil, J.A. (2001). Construction of a xylanase-producing strain of *Brevibacterium lactofermentum* by stable integration of an engineered *xysA* gene from *Streptomyces halstedii* JM8. *Appl Environ Microbiol* 67(12)**,** 5425-5430.

Díaz, M., Esteban, A., Fernández-Ábalos, J.M., and Santamaría, R.I. (2005). The high-affinity phosphate-binding protein PstS is accumulated under high fructose concentrations and mutation of the corresponding gene affects differentiation in *Streptomyces lividans*. *Microbiology* 151(Pt 8)**,** 2583-2592.

Kuhstoss, S., Richardson, M.A., and Rao, R.N. (1991). Plasmid cloning vectors that integrate site-specifically in *Streptomyces* spp. *Gene* 97(1)**,** 143-146. doi: 10.1016/0378-1119(91)90022-4.

Paget, M.S., Leibovitz, E., and Buttner, M.J. (1999). A putative two-component signal transduction system regulates *sigmaE*, a sigma factor required for normal cell wall integrity in *Streptomyces coelicolor A3*(2). *Mol Microbiol* 33(1)**,** 97-107.

Tong, Y., Charusanti, P., Zhang, L., Weber, T., and Lee, S.Y. (2015). CRISPR-Cas9 Based Engineering of Actinomycetal Genomes. *ACS synthetic biology*. doi: doi: 10.1021/acssynbio.5b00038.

Supplementary Figures

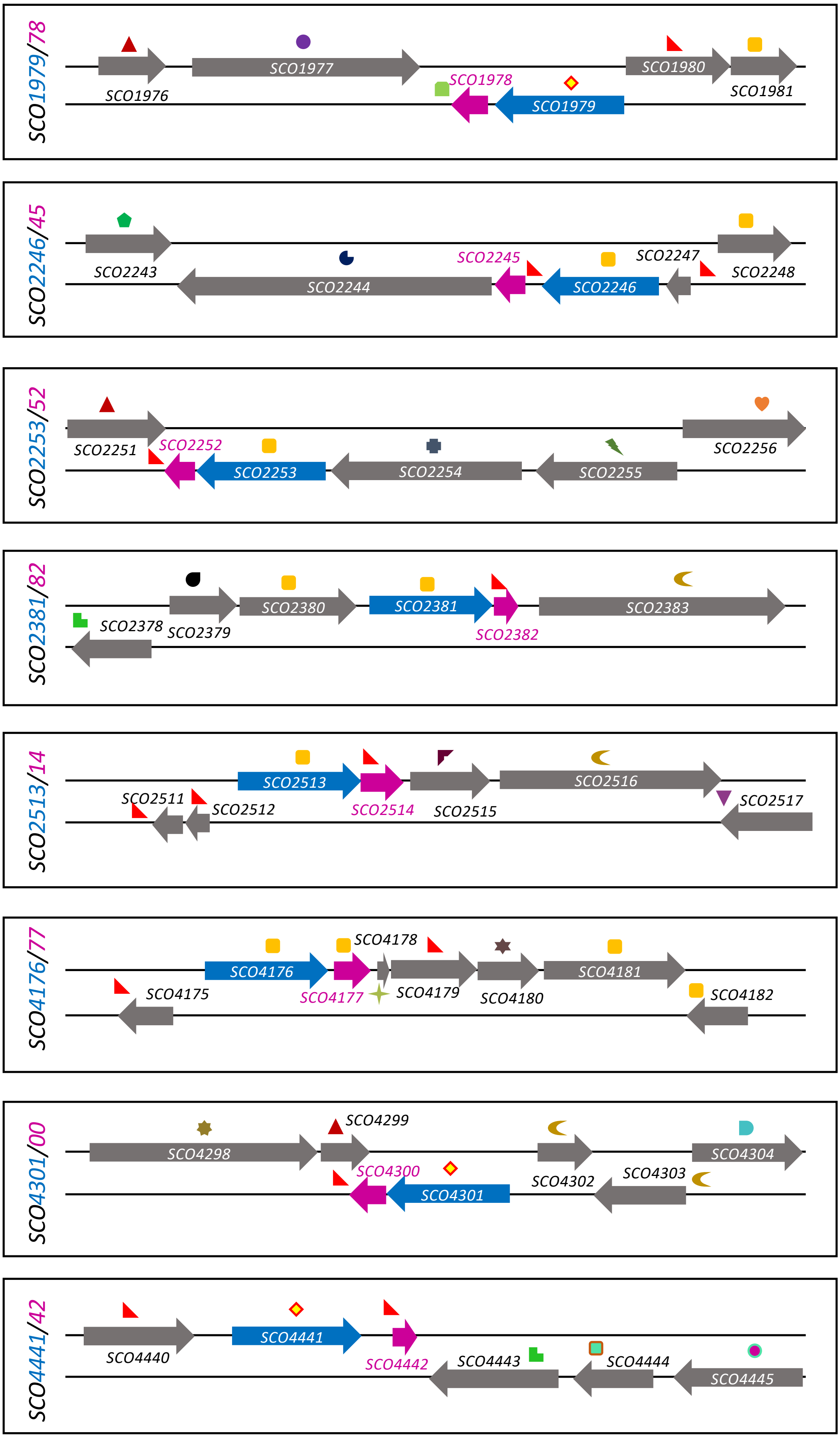

Supplementary Figure 1. Location of genes encoding the XRE/DUF397 systems and their flanking genes in the genome of S. coelicolor. ▲ Putative integral membrane protein; ● Putative small subunit of glutamate synthase; ◣ Hypothetical protein; ■ Hypothetical conserved protein; ■ Putative AbaA-like protein; ◇ Putative DNA-binding protein; ⬟ Putative transcriptional regulator of the TetR family; Probable serine/threonine protein kinase; ♥ 3-methyl-2-oxobutanoate hydroxymethyltransferase; ✚ Putative transmembrane efflux protein; Putative membrane protein; Putative acetyltransferase; Putative secreted protein; Putative transcriptional regulator of the merR family; Putative lipoprotein; ▼ Putative two-component system response regulator; ✦ Putative Small Membrane Protein; ✶ Putative iron uptake regulatory protein; Putative oxidoreductase; ✸ Putative carboxylesterase; ▣ Putative glutathione peroxidase; ◉ Putative transcriptional regulator.

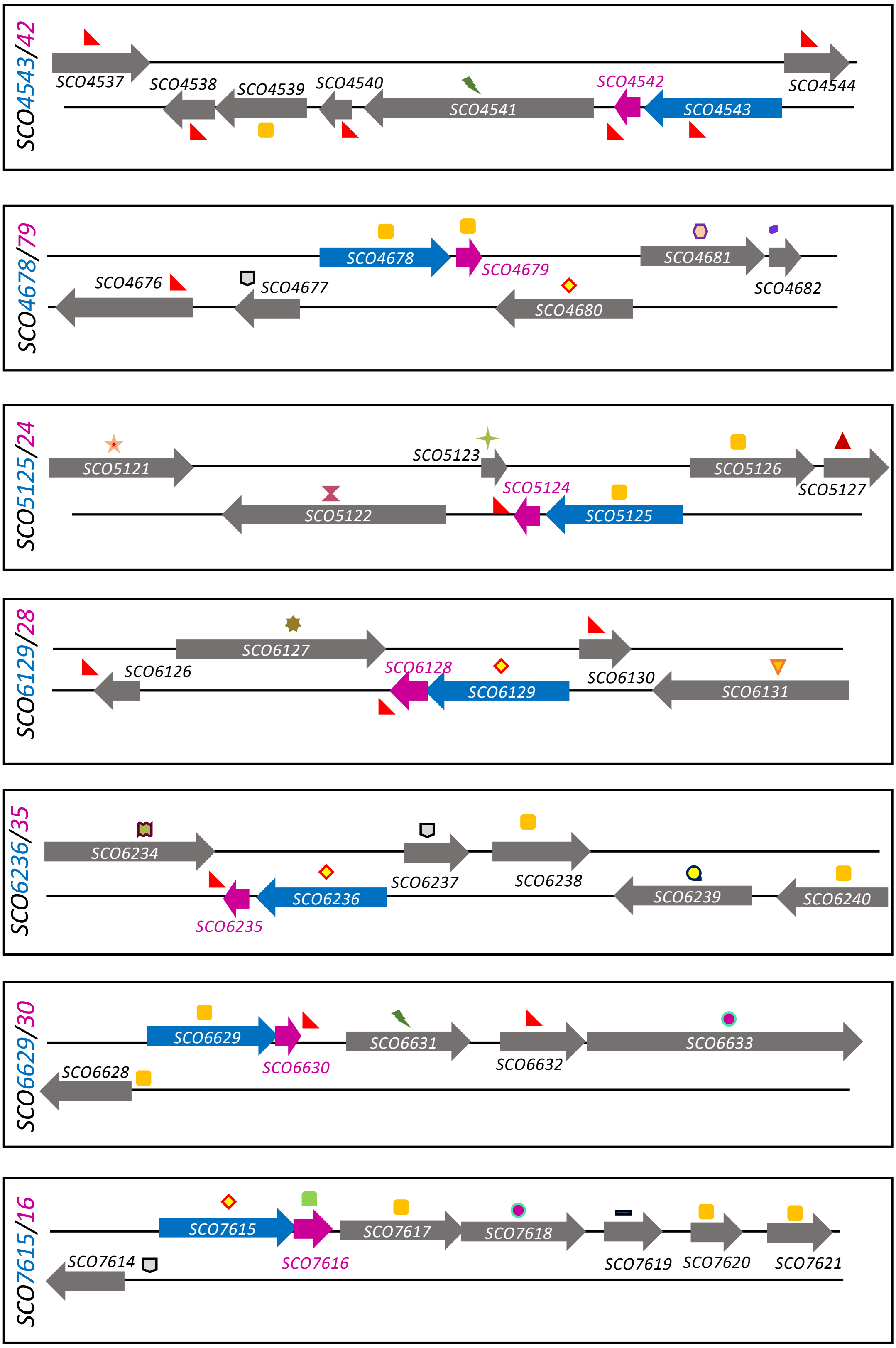

Supplementary Figure 2. Location of genes encoding the XRE/DUF397 systems and their flanking genes in the genome of S. coelicolor. Hypothetical protein; Conserved hypothetical protein; Putative membrane protein; Putative regulatory protein; Putative DNA-binding protein; Putative short chain dehydrogenase; Putative tautomerase; Putative peptide transport system ATP-binding protein; Putative peptidase; Putative Small Membrane Protein; Putative integral membrane protein; Putative carboxylesterase; Putative carboxypeptidase; Secreted beta-mannosidase; Putative sigma factor; Putative transcriptional regulator; Putative AbaA-like protein; Putative anti sigma factor antagonist.

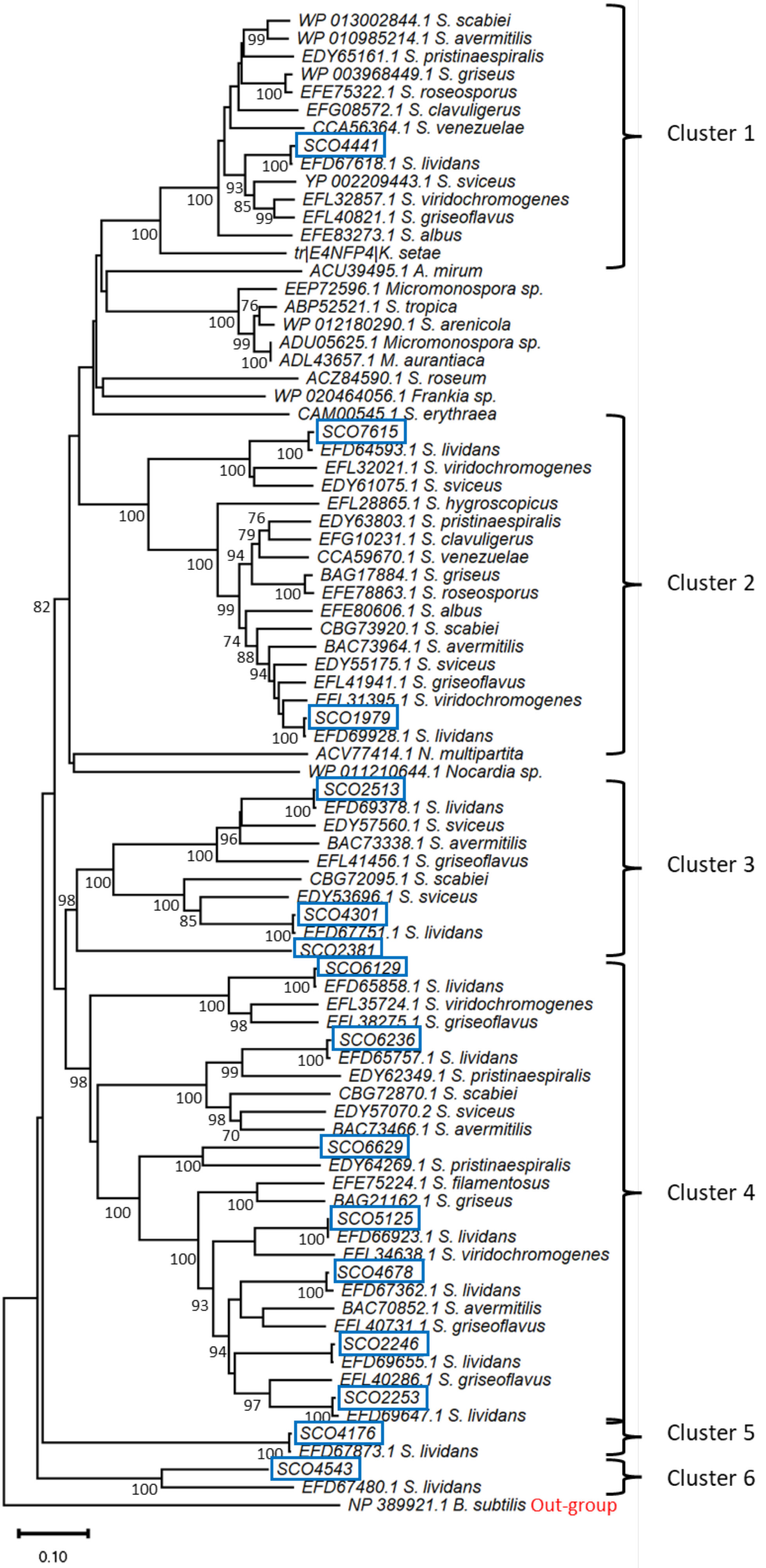

Supplementary Figure 3. Phylogenetic tree of *S. coelicolor* XRE genes and their orthologues in the phylum actinobacteria, identity ≥ 70 %. MEGA11 program Neighbor-Joining Bootstrap method 1000 copies was used. XRE genes studied.

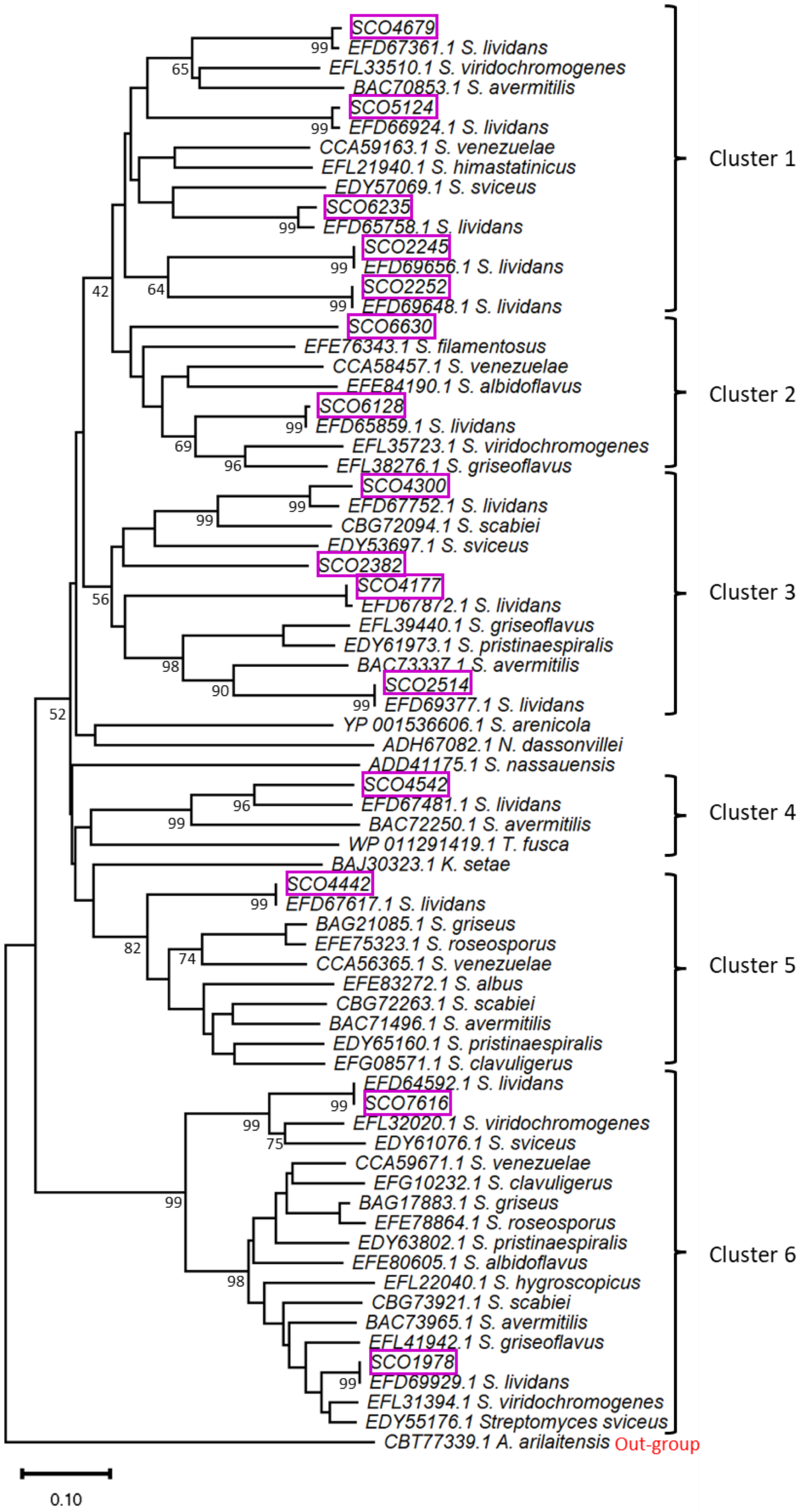

Supplementary Figure 4. Phylogenetic tree of *S. coelicolor* DUF397 genes and their orthologues in the phylum actinobacteria ≥ 70 % identity. MEGA11 program Neighbor-Joining Bootstrap method 1000 copies was used. ▇ DUF397 genes studied.

Percentage of identity of the proteins XRE

	SCO1979	SCO2246	SCO2253	SCO2381	SCO2513	SCO4176	SCO4301	SCO4441	SCO4543	SCO4678	SCO5125	SCO6129	SCO6236	SCO6629	SCO7615
1: SCO1979	**100.00**	27.80	24.72	28.57	23.33	22.88	28.78	34.04	31.21	27.72	26.97	27.51	26.10	27.99	54.23
2: SCO2246	27.80	**100.00**	76.36	33.62	26.97	27.69	28.75	25.82	26.78	68.36	66.67	29.07	31.37	42.57	27.87
3: SCO2253	24.72	76.36	**100.00**	30.65	25.47	29.10	26.69	25.93	25.28	70.92	67.96	31.34	32.38	45.82	27.88
4: SCO2381	28.57	33.62	30.65	**100.00**	35.06	26.47	36.13	27.17	24.71	31.80	31.03	31.56	30.60	32.06	28.57
5: SCO2513	23.33	26.97	25.47	35.06	**100.00**	28.47	42.55	27.88	24.25	28.84	26.59	29.37	28.15	30.22	24.44
6: SCO4176	22.88	27.69	29.10	26.47	28.47	**100.00**	25.09	30.00	27.24	25.75	27.24	27.41	26.01	28.62	28.41
7: SCO4301	28.78	28.75	26.69	36.13	42.55	25.09	**100.00**	34.07	25.75	28.57	30.08	27.61	27.84	27.72	26.20
8: SCO4441	34.04	25.82	25.93	27.17	27.88	30.00	34.07	**100.00**	33.57	27.24	28.94	26.18	26.01	25.66	34.84
9: SCO4543	31.21	26.78	25.28	24.71	24.25	27.24	25.75	33.57	**100.00**	24.53	28.30	23.60	20.82	22.56	29.89
10: SCO4678	27.72	68.36	70.92	31.80	28.84	25.75	28.57	27.24	24.53	**100.00**	66.31	30.14	29.54	43.27	26.39
11: SCO5125	26.97	66.67	67.96	31.03	26.59	27.24	30.08	28.94	28.30	66.31	**100.00**	30.66	31.21	48.00	25.83
12: SCO6129	27.51	29.07	31.34	31.56	29.37	27.41	27.61	26.18	23.60	30.14	30.66	**100.00**	33.57	35.74	25.64
13: SCO6236	26.10	31.37	32.38	30.60	28.15	26.01	27.84	26.01	20.82	29.54	31.21	33.57	**100.00**	36.10	26.91
14: SCO6629	27.99	42.57	45.82	32.06	30.22	28.62	27.72	25.66	22.56	43.27	48.00	35.74	36.10	**100.00**	29.21
15: SCO7615	54.23	27.87	27.88	28.57	24.44	28.41	26.20	34.84	29.89	26.39	25.83	25.64	26.91	29.21	**100.00**

Percentage of identity of the proteins DUF397

	SCO1978	SCO2245	SCO2252	SCO2382	SCO2514	SCO4177	SCO4300	SCO4442	SCO4542	SCO4679	SCO5124	SCO6128	SCO6235	SCO6630	SCO7616
1: SCO1978	**100.00**	27.12	26.98	31.75	31.33	26.32	29.33	35.48	29.51	26.42	23.21	28.12	23.53	32.76	62.20
2: SCO2245	27.12	**100.00**	56.52	43.90	35.38	42.11	39.68	42.86	25.58	42.86	46.97	46.55	51.61	41.67	26.92
3: SCO2252	26.98	56.52	**100.00**	41.46	37.68	34.43	31.34	38.10	26.67	55.56	46.97	44.83	53.23	37.50	21.43
4: SCO2382	31.75	43.90	41.46	**100.00**	42.19	51.56	55.36	43.55	37.93	39.02	53.66	52.73	42.11	43.86	34.92
5: SCO2514	31.33	35.38	37.68	42.19	**100.00**	44.32	47.67	41.27	32.26	36.51	40.00	46.75	39.51	35.82	27.50
6: SCO4177	26.32	42.11	34.43	51.56	44.32	**100.00**	51.85	38.10	37.10	36.84	38.60	54.17	42.11	46.27	28.95
7: SCO4300	29.33	39.68	31.34	55.36	47.67	51.85	**100.00**	45.45	36.21	38.10	44.44	47.37	36.25	36.36	28.00
8: SCO4442	35.48	42.86	38.10	43.55	41.27	38.10	45.45	**100.00**	34.48	35.71	40.48	45.28	44.64	48.21	33.87
9: SCO4542	29.51	25.58	26.67	37.93	32.26	37.10	36.21	34.48	**100.00**	27.91	25.58	44.23	33.93	40.00	26.23
10: SCO4679	26.42	42.86	55.56	39.02	36.51	36.84	38.10	35.71	27.91	**100.00**	50.79	46.55	56.45	39.58	25.00
11: SCO5124	23.21	46.97	46.97	53.66	40.00	38.60	44.44	40.48	25.58	50.79	**100.00**	46.55	50.00	47.92	34.62
12: SCO6128	28.12	46.55	44.83	52.73	46.75	54.17	47.37	45.28	44.23	46.55	46.55	**100.00**	53.25	50.00	29.69
13: SCO6235	23.53	51.61	53.23	42.11	39.51	42.11	36.25	44.64	33.93	56.45	50.00	53.25	**100.00**	44.78	25.00
14: SCO6630	32.76	41.67	37.50	43.86	35.82	46.27	36.36	48.21	40.00	39.58	47.92	50.00	44.78	**100.00**	32.76
15: SCO7616	62.20	26.92	21.43	34.92	27.50	28.95	28.00	33.87	26.23	25.00	34.62	29.69	25.00	32.76	**100.00**

Percent Identity Matrix - created by Clustal2.1

Supplementary Figure 5 identity of the different XRE/DUF397 proteins from *S. coelicolor*.

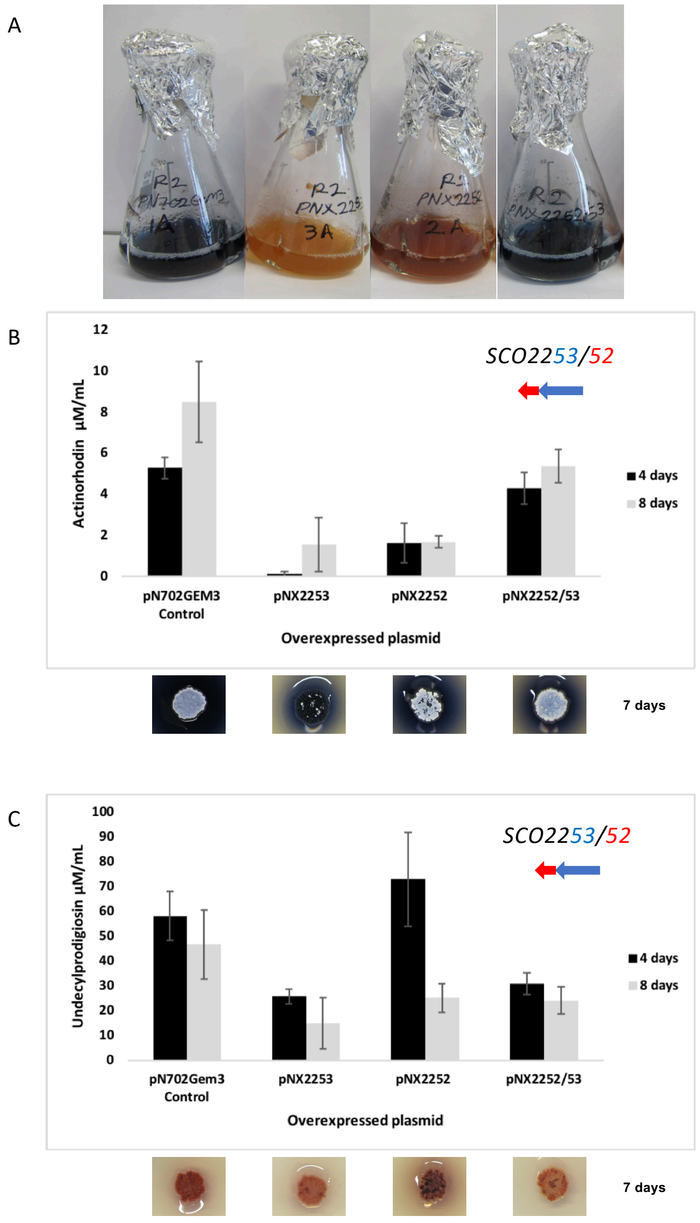

Supplementary Fig 6: **Qualitative and quantitative antibiotic production of the SCO2253/52 overexpression *S. coelicolor* strains.** A) Phenotypes of the LB liquid cultures of *S. coelicolor* harbouring pN702Gem3 (control), pNX22253, pNX2252, or pNX2252/53 plasmids at four days; B) Quantification of ACT production of the cultures (upper part) and comparison with the phenotypes obtained on solid LB médium (lower part- data from figure 3); C) Quantification of RED production of the cultures (upper part) and comparison with the phenotypes obtained on PGA solid medium (lower part- data from figure 3). The experiments were made by triplicate.

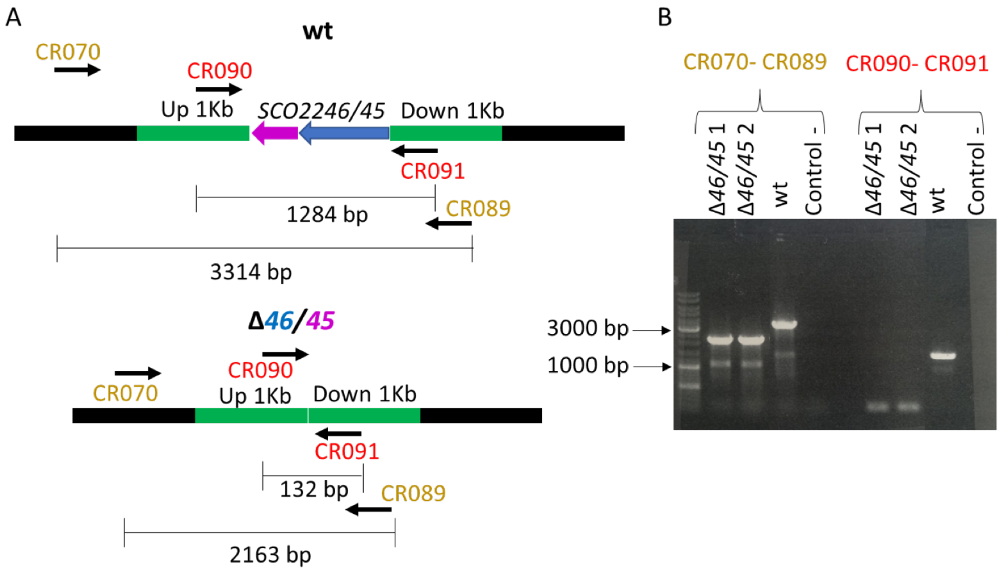

Supplementary Figure 7. ΔSCO2246/45 mutant verification. (**A**) Schema of the primer design used to verify the Δ*46/45* mutant by PCR. ■ XRE gene; ■ DUF397 gene; ■ homologous DNA template; ■ *S. coelicolor* M145 genome. (**B**) Agarose gel with the corresponding wild type (wt) and mutant bands.

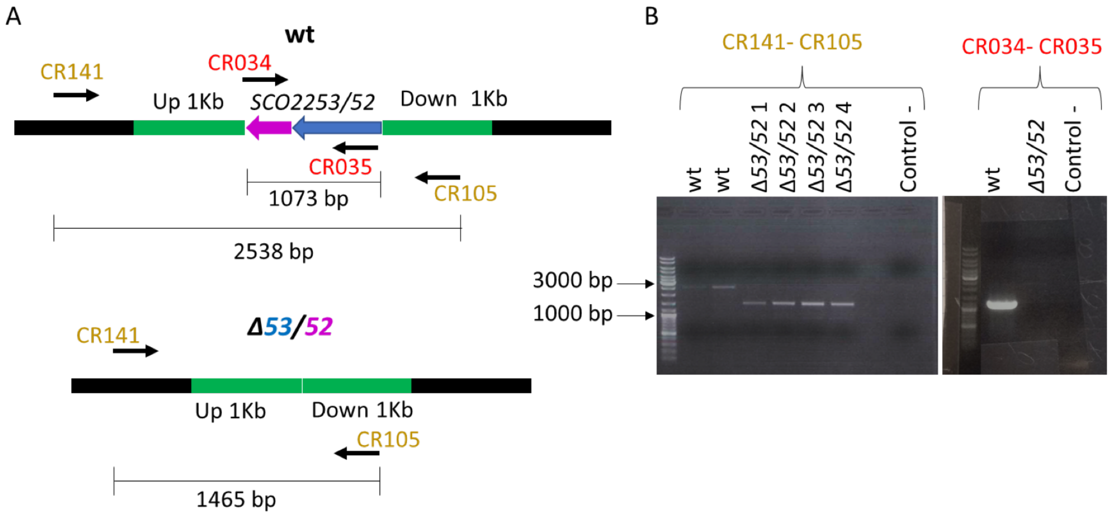

Supplementary Figure 8. ***ΔSCO2253/52*** **mutant verification**. (**A**) Schema of the primer design used to verify the *Δ53/52* mutant by PCR. ■ XRE gene; ■ DUF397 gene; ■ homologous DNA template; ■ *S. coelicolor* M145 genome. (**B**) Agarose gel with the corresponding wild type (wt) and mutant bands.

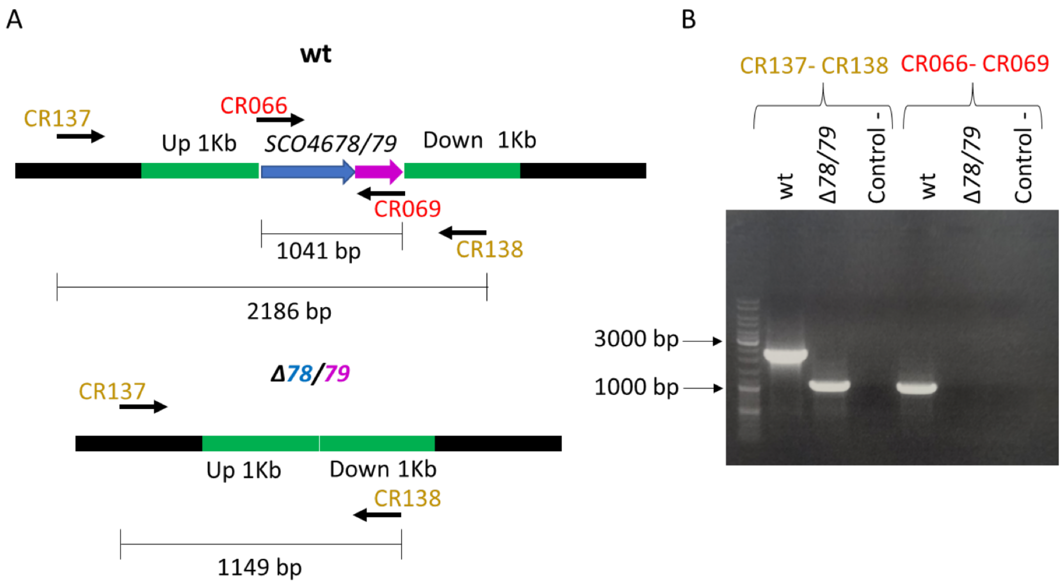

Supplementary Figure 9. *ΔSCO4678/79* mutant verification. (**A**) Schema of the primer design used to verify the *Δ78/79* mutant by PCR. ■ XRE gene; ■ DUF397 gene; ■ homologous DNA template; ■ *S. coelicolor* M145 genome. (**B**) Agarose gel with the corresponding wild type (wt) and mutant bands.

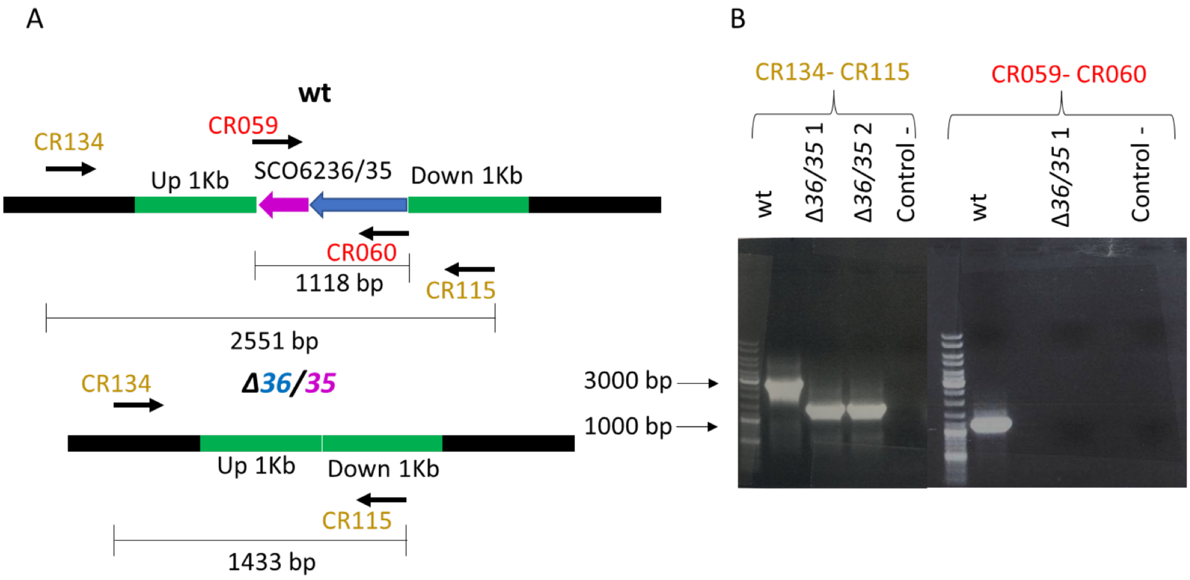

Supplementary Figure 10. *ΔSCO6236/35* mutant verification. (**A**) Schema of the primer design used to verify the Δ*36/35* mutant by PCR. ■XRE gene; ■DUF397 gene; ■ homologous DNA template; ■ *S. coelicolor* M145 genome. (**B**) Agarose gel with the corresponding wild type (wt) and mutant bands.

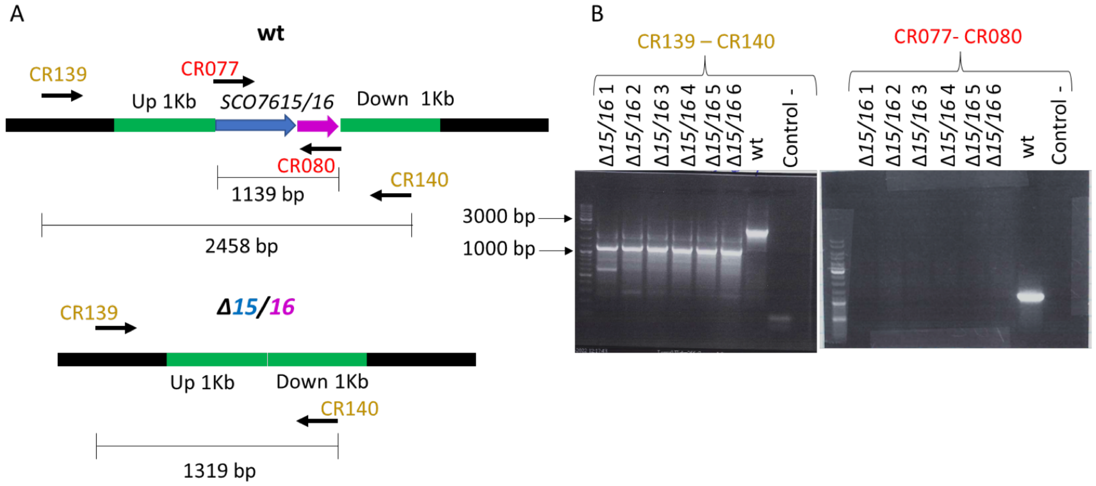

Supplementary Figure 11. *ΔSCO7615/16* mutant verification. (**A**) Schematic of the primer design, used to verify the Δ*15/16* mutant by PCR. XRE gene; DUF397 gene; homologous DNA template; *S. coelicolor* M145 genome. (**B**) Agarose gel with the corresponding wild type (wt) and mutant bands.
